# Supplementary material for: China’s recycling potential of large-scale public transport vehicles and its implications
Source: Commun Eng. 2023 Aug 7;2:56. doi: 10.1038/s44172-023-00106-y (PMC10956039; doi:10.1038/s44172-023-00106-y)
Supplement: Supplementary file 2 — Supplementary Information [file 44172_2023_106_MOESM2_ESM.pdf]

Supplementary Information

China’s recycling potential of large-scale public transport vehicles and its implications

Xin Xiong, Xianlai Zeng✉, Zhengyang Zhang, Robert Pell, Kazuyo Matsubae & Zhaoji Hu

✉email: xlzeng@tsinghua.edu.cn

Contents

Supplementary Table1 Categories of large-scale public transport vehicle S1

Supplementary Table 2 Data demands for various approaches S2

Supplementary Table 3 Production amounts of LPTV in China (unit)................................S3

Supplementary Table 4 Possession amounts of typical LPTV in China (unit)......................S4

Supplementary Table 5 Mass of each unit LPTV (t)..S5

Supplementary Fig. 1 Estimation of China’s WLPTV from 2000 to 2050 ....S6

Supplementary Table 6 The possession amounts of railway equipment in developed countries (unit). S7

Supplementary Table 7 LPTV possession per capita in countries S7

Supplementary Table 8 China's population in the year 2000-2020 ....S8

Supplementary Table 9 China's per capita GDP in the year 2000-2020 (US$)....................S8

Supplementary Fig. 2 Relationship of carbon footprint during virgin and recycled material production S9

Supplementary Table 10 Amounts of generated and potentially saved CO_2 emissions from replacing virgin with recycled materials...S9

Supplementary Fig. 3 Estimated typical materials carbon footprint of China’s LPTV manufacturing industry in 2000-2050..S9

Supplementary Table 11 Typical material composition of LPTV S10

Supplementary Fig. 4 Function of Weibull distribution S11

Supplementary Table 12 Parameters of Weibull lifespan distribution for LPTV...............S11

Supplementary Table 13 Obsolescence probability distribution of life-span for six categories of LPTV S12

Supplementary Fig. 5 Data regression of possession amount of LPTV. S13

Supplementary Table 14 Market prices of Fe, Al, Nd, and Ti (US$/t) S14

Supplementary Fig. 6 Monte Carlo Simulation for RPC, RL, RW, HST, LMA and GAA........S36

Supplementary Note 1 The abbreviations and acronyms of main vocabulary S37

Supplementary references S38

Supplementary Table1 Categories of large-scale public transport vehicle [1]

Typical large-scale public transport vehicle	Categories
Railway equipment	Railway locomotives (RL)
	Railway passenger car (RPC)
	Railway wagon (RW)
	High-speed trains (HST)
Aviation equipment	Large and medium aircraft (LMA)
	General aviation aircraft (GAA)

Supplementary Table 2 Data demands for various approaches

Approach	Other designation	Sales (Production, import and export)	Lifetime distribution	Possession	Historical collected/ generation amount	Saturation factor	Substitution effect	Influential factors	Econometric indicators
Market supply method[2]	Classic market supply method /sales/simple delay	√	√						
Market supply A method[3, 4]	Distribution delay	√	√						
Stanford method[5]		√	√			√			
Carnegie Mellon method[6]		√	√						
Consumption and use approach[3]	Approximation/ estimate formula/batch leaching		√	√					
Time-step method[7, 8]		√		√	√				
MFA method[9]		√		√					
Use-phase analysis[10]			√	√			√		
Time-series model[11]					√				
Factor model[12]	Projection				√			√	
Direct waste analysis[12]	Waste facility record compilation/disposal related analysis				√				
Possession coefficient method[13, 14]			√	√					

Supplementary Table 3 Production amounts of LPTV in China (unit)

Year	HST	RL	RPC	RW
1990		655	1866	18600
1991		706	1674	18500
1992		798	1652	21600
1993		922	1847	29000
1994		992	1837	37600
1995		974	2395	37300
1996		1050	2616	32800
1997		1069	2535	31200
1998		278	1576	23500
1999		300	1778	18600
2000	176	243	3244	27300
2001	93	303	3273	30700
2002	14	336	2856	31300
2003	2	323	1525	31200
2004	11	442	1867	31700
2005	5	838	2001	39200
2006	18	1000	2143	39300
2007	65	1000	2425	44500
2008	264	1100	2930	57300
2009	39	1800	7107	42800
2010	478	2571	7450	48100
2011	1580	2530	6853	66900
2012	1983	1622	7562	59200
2013	1898	1436	3631	56700
2014	3952	1658	3438	34400
2015	3798	1979	2572	27300
2016	3474	1292	1303	24600
2017	2600	1500	330	51800
2018	2724	1461	914	50500
2019	2117	1319	1641	60300

Source: https://navi.cnki.net/knavi/yearbooks/YZGJN/detail

Supplementary Table 4 Possession amounts of typical LPTV in China (unit)

Year	RL	RPC	RW	HST	LMA	GAA
1987	12729	23474	328146			
1988	13163	24917	340299			
1989	13366	26304	353041			
1990	13592	27261	364966			217
1991	13906	27612	370054		139	196
1992	14083	28464	373233		211	287
1993	14397	29395	390097		280	273
1994	14694	31018	415919		316	392
1995	15146	32404	432731		330	306
1996	15403	34516	448280		356	302
1997	15335	35171	442501		397	285
1998	15176	34246	439326		443	278
1999	14480	34535	436236		445	285
2000	14472	35989	439943		462	301
2001	14955	37214	449921		486	296
2002	15159	37942	446707		525	335
2003	15456	38972	503868		580	343
2004	16066	39766	520101		675	365
2005	16547	40328	541824		785	383
2006	16904	40945	558483		921	457
2007	17311	42471	571078		1050	457
2008	17336	43215	584961	1810	1155	484
2009	17825	47436	594388	2850	1297	555
2010	18349	50391	622284	4080	1453	606
2011	19590	52838	644677	6816	1601	1124
2012	19625	55764	664333	8656	1769	1320
2013	19686	56841	715492	10464	1985	1519
2014	21096	60945	716578	13969	2218	1798
2015	21366	67706	768516	17648	2499	1904
2016	21453	70872	764783	20686	2789	2096
2017	21420	72262	808736	23480	3120	2297
2018	21482	73335	839213	26048	3452	2495
2019	22000	74848	877134	29319	3626	2707
2020	22000	76033	912735	31340	3701	2892
2021	*22500*	*81189*	*854574*	*38400*	*4077*	*3322*
2022	*22711*	*84117*	*869061*	*44256*	*4395*	*3624*
2023	*22907*	*87075*	*882738*	*50717*	*4718*	*3927*
2024	*23089*	*90057*	*895619*	*57760*	*5043*	*4229*
2025	*23258*	*93059*	*907725*	*65339*	*5368*	*4525*
2026	*23415*	*96075*	*919079*	*73382*	*5690*	*4813*

2027	*23560*	*99099*	*929707*	*81791*	*6007*	*5088*
2028	*23694*	*102127*	*939637*	*90449*	*6315*	*5349*
2029	*23818*	*105152*	*948901*	*99221*	*6613*	*5593*
2030	*23932*	*108170*	*957528*	*107968*	*6899*	*5820*
2031	*24038*	*111175*	*965551*	*116550*	*7172*	*6028*
2032	*24135*	*114162*	*973001*	*124838*	*7429*	*6217*
2033	*24225*	*117126*	*979912*	*132721*	*7671*	*6388*
2034	*24307*	*120061*	*986315*	*140112*	*7897*	*6542*
2035	*24383*	*122962*	*992240*	*146946*	*8107*	*6678*
2036	*24453*	*125827*	*997718*	*153188*	*8300*	*6799*
2037	*24517*	*128649*	*1002777*	*158823*	*8478*	*6906*
2038	*24576*	*131425*	*1007446*	*163857*	*8640*	*6999*
2039	*24630*	*134151*	*1011751*	*168313*	*8788*	*7081*
2040	*24680*	*136823*	*1015718*	*172225*	*8922*	*7152*
2041	*24725*	*139440*	*1019371*	*175635*	*9043*	*7214*
2042	*24767*	*141997*	*1022733*	*178589*	*9152*	*7268*
2043	*24805*	*144492*	*1025825*	*181133*	*9250*	*7314*
2044	*24840*	*146924*	*1028667*	*183314*	*9337*	*7354*
2045	*24872*	*149290*	*1031278*	*185177*	*9416*	*7388*
2046	*24902*	*151590*	*1033676*	*186762*	*9486*	*7417*
2047	*24929*	*153820*	*1035878*	*188107*	*9548*	*7443*
2048	*24953*	*155982*	*1037898*	*189245*	*9603*	*7464*
2049	*24976*	*158075*	*1039751*	*190207*	*9652*	*7483*
2050	*24997*	*160097*	*1041451*	*191017*	*9696*	*7499*

Note: The italic data indicates the estimated value.

Source: https://data.stats.gov.cn/easyquery.htm?cn=C01&zb=A060401&sj=2020;
https://navi.cnki.net/knavi/yearbooks/YZGTD/detail

Supplementary Table 5 Mass of each unit LPTV (t)[15, 16]

Type	LMA	GAA	HST	RL	RPC	RW
Mean	130	42	450	130	750	800
Range	90–177	38–48	420–510	126–132	700–800	750–850

Note: The data fits for beta distribution.

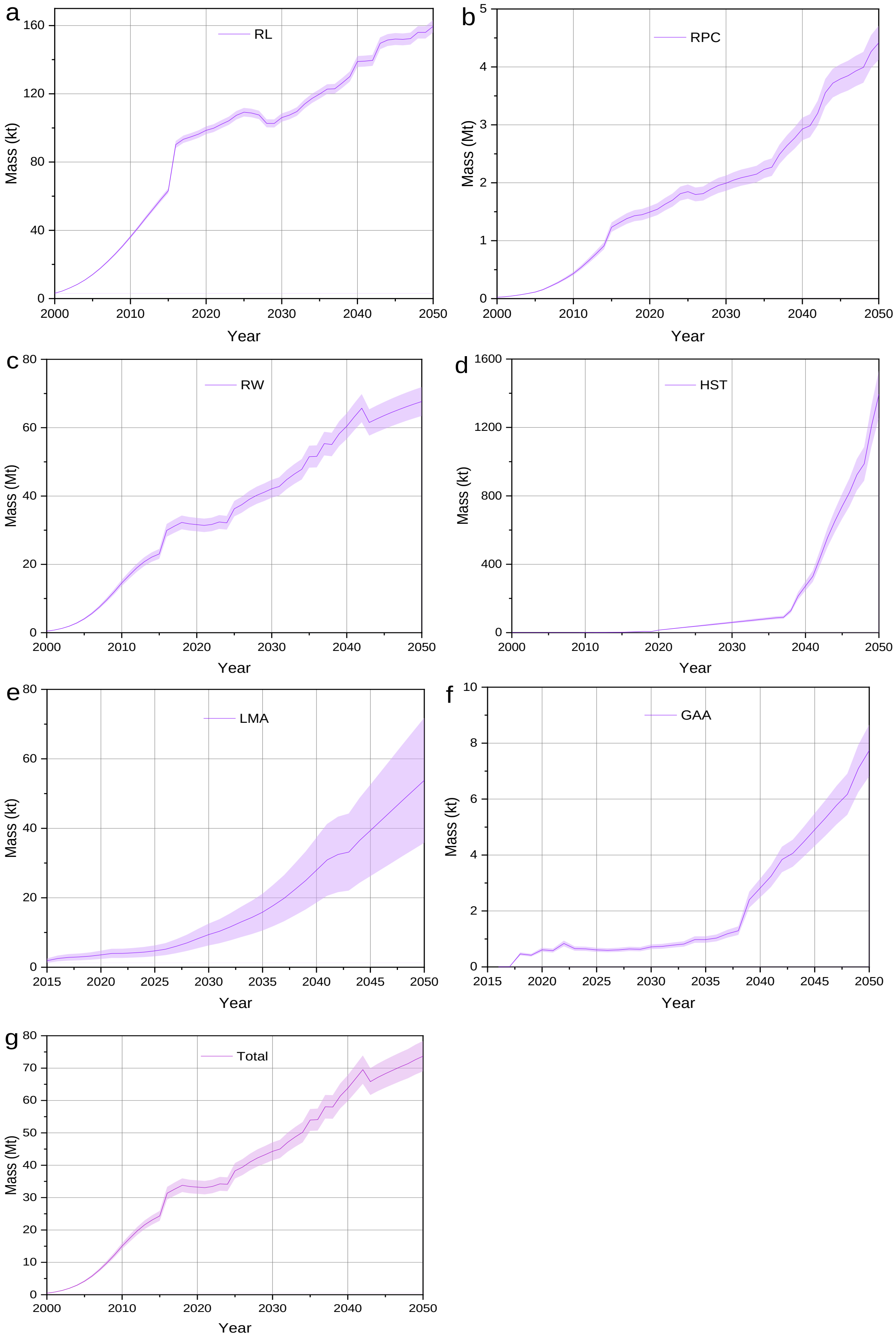

Supplementary Fig. 1 Estimation of China's WLPTV from 2000 to 2050

Supplementary Table 6 The possession amounts of railway equipment in developed countries (unit).

Country	Categories	2017	2018	2019
Japan	Railway locomotives	908	908	102
	Railway passenger car	25047	25047	25088
	Railway wagon	15714	15714	15714
	High-speed trains	15014	16332	15869
Germany	Railway locomotives	4115	4115	3863
	Railway passenger car	18055	18055	17888
	Railway wagon	80608	80608	79531
	High-speed trains	12720	16785	16987
The United Kingdom	Railway locomotives	249	249	244
	Railway passenger car	12286	12286	12286
	Railway wagon	8284	8284	8284
	High-speed trains	11098	11107	11133

Source: https://navi.cnki.net/knavi/yearbooks/YZGJN/detail

Supplementary Table 7 LPTV possession per capita in countries

Country	Categories	Mass (t)	per capita possession (kg)
China	Railway locomotive (2050)	3,249,610	2.5
	Railway passenger car (2050)	120,072,750	91.6
	Railway wagon (2050)	833,160,800	635.3
	High-speed train (2050)	85,957,650	65.5
Japan	Railway locomotives	13,260	0.1
	Railway passenger car	18,816,000	148.6
	Railway wagon	12,571,200	99.3
	High-speed trains	7,141,050	56.4
Germany	Railway locomotives	502,190	6.0
	Railway passenger car	13,416,000	161.5
	Railway wagon	63,624,800	765.7
	High-speed trains	7,644,150	92.0
United Kingdom	Railway locomotives	31,720	0.5
	Railway passenger car	9,214,500	137.9
	Railway wagon	6,627,220	99.2
	High-speed trains	5,009,850	74.9

Note: per capita possession= possession of railway equipment/ population. According to the statistics of the World Bank, the population of Japan, Germany and the United Kingdom are 126,633,000, 83,092,962 and 66,836,327 respectively, and the population forecast of China in 2050 is 1,311,400,000[17].

Supplementary Table 8 China's population in the year 2000-2020

Year	Population	Year	Population
2000	1,262,645,000	2011	1,344,130,000
2001	1,271,850,000	2012	1,350,695,000
2002	1,280,400,000	2013	1,357,380,000
2003	1,288,400,000	2014	1,364,270,000
2004	1,296,075,000	2015	1,371,220,000
2005	1,303,720,000	2016	1,378,665,000
2006	1311020,000	2017	1,386,395,000
2007	1,317,885000	2018	1,392,730,000
2008	1,324,655,000	2019	1,397,715,000
2009	1,331,260,000	2020	1,402,112,000
2010	1,337,705,000		

Source: https://data.worldbank.org.cn/indicator/SP.POP.TOTL?locations=CN

Supplementary Table 9 China's per capita GDP in the year 2000-2020 (US$)

Year	Per capita GDP	Year	Per capita GDP
2000	959	2011	5618
2001	1053	2012	6317
2002	1149	2013	7051
2003	1289	2014	7679
2004	1509	2015	8067
2005	1753	2016	8148
2006	2099	2017	8879
2007	2694	2018	9977
2008	3468	2019	10217
2009	3832	2020	10500
2010	4550		

Source: https://data.worldbank.org.cn/indicator/NY.GDP.PCAP.CD?locations=CN

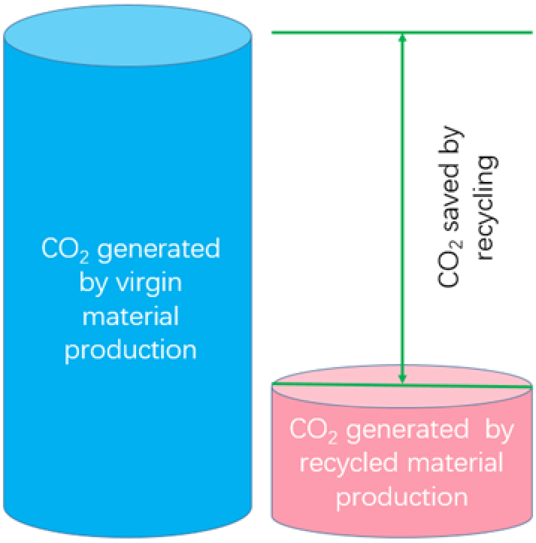

Supplementary Fig. 2 Relationship of carbon footprint during virgin and recycled material production

Supplementary Table 10 Amounts of generated and potentially saved CO_2 emissions from replacing virgin with recycled materials[18, 19, 20]

Metal	Virgin material production ($t\ CO_2/t$)		Recycled material production ($t\ CO_2/t$)		Saved by recycling ($kg\ CO_2/kg$)	
	Mean	Error	Mean	Error	Mean	Error
Fe	1.76	±1.3	0.7	±0.32	1.06	±0.98
Al	5.83	±1.94	0.6	±0.31	5.23	±1.63
Ti	1.25	±0.5	0.44	±0.16	0.81	±0.34
Nd	17	±9	10	±8	7	±2

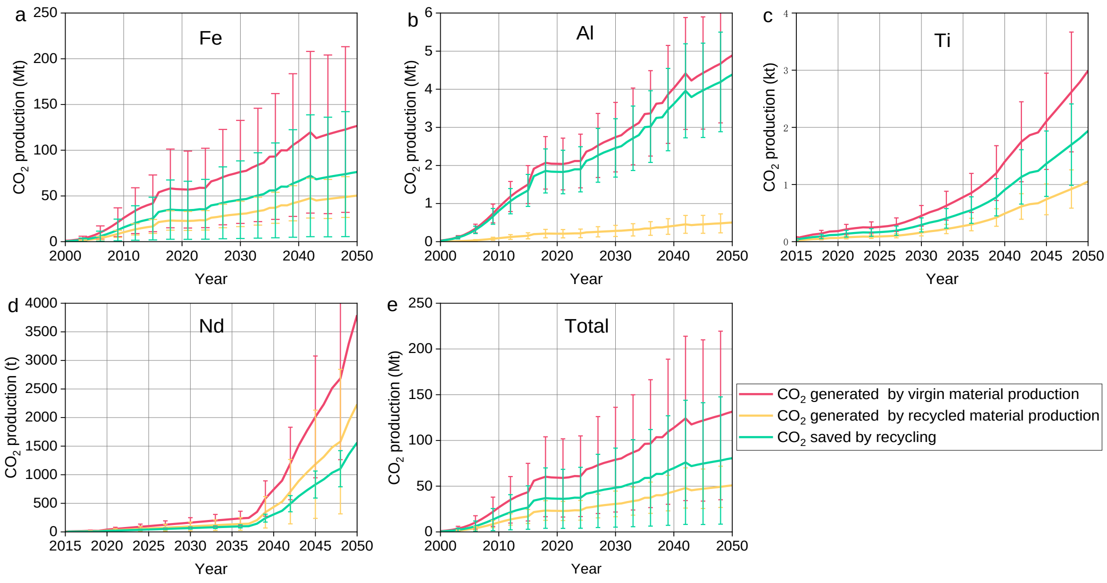

Supplementary Fig. 3 Estimated typical materials carbon footprint of China's LPTV manufacturing industry in 2000-2050: **a** Fe. **b** Al. **c** Ti. **d** Nd. **e** Total.

Supplementary Table 11 Typical material composition of LPTV[16, 21]

a LMA (w.t. %)

Material	Min	Average	Max
Al	55	70	85
Fe	9	12	15
Nd	-	-	-
Ti	2	4	6

b GAA (w.t. %)

Material	Min	Average	Max
Al	70	80	90
Fe	9	13	16
Nd	-	-	-
Ti	3	5	7

c HST (w.t. %)

Material	Min	Average	Max
Al	1	2	3
Fe	82	90	95
Nd	0.01	0.016	0.022
Ti	0	0	0

d RL (w.t. %)

Material	Min	Average	Max
Al	0.5	1	1.5
Fe	95	96	98
Nd	0	0	0
Ti	0	0	0

e RPC (w.t. %)

Material	Min	Average	Max
Al	1	2	3
Fe	92	95	97
Nd	0	0	0
Ti	0	0	0

f RW (w.t. %)

Material	Min	Average	Max
Al	0.5	1	1.5
Fe	97	98	99
Nd	0	0	0
Ti	0	0	0

Note: The data fits for beta distribution.

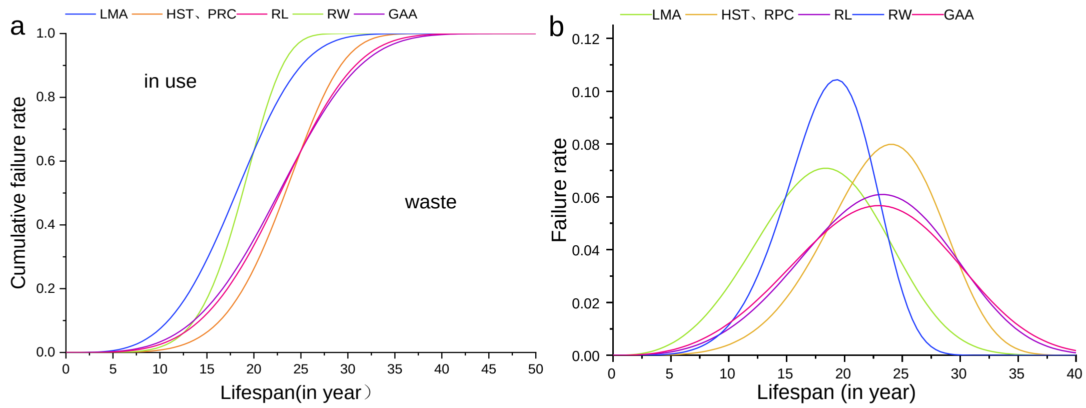

Supplementary Fig. 4 Function of Weibull distribution: **a** cumulative distribution function; **b** probability density function

Note: The LPTV cumulative and annual failure rates are shown in Supplementary Fig. 4a and Fig. 4b, respectively. The two zones of in-use and waste are located above and below the function curve, respectively. Therefore, supplementary Fig. 4a can be employed to define the cumulative obsolete amount and stocked resources of various WLPTV, and supplementary Fig. 4b can be utilized to determine the yearly obsolete amount and stocked resources of various WLPTV[22].

Supplementary Table 12 Parameters of Weibull lifespan distribution for LPTV[23]

Parameter	RL	RPC	RW	HST	LMA	GAA
η (scale)	25	25	20	25, [20-30]	20, [15-25]	25, [20-30]
β (shape)	4, [3-5]	5.33, [5-6]	5.58, [5-6]	5.33, [5-6]	3.7, [3-4]	3.7, [3-4]

Note: [] indicates the range

Supplementary Table 13 Obsolescence probability distribution of life-span for six categories of LPTV

Lifespan (year)	LMA	GGA	HST	RL	RPC	RW
30	0.006	0.034	0.033	0.035	0.033	0.000
29	0.011	0.039	0.045	0.041	0.045	0.001
28	0.016	0.044	0.056	0.047	0.056	0.002
27	0.022	0.048	0.066	0.052	0.066	0.005
26	0.030	0.052	0.074	0.056	0.074	0.012
25	0.038	0.054	0.078	0.059	0.078	0.024
24	0.047	0.056	0.080	0.061	0.080	0.040
23	0.056	0.057	0.078	0.061	0.078	0.060
22	0.064	0.056	0.074	0.060	0.074	0.078
21	0.071	0.055	0.068	0.058	0.068	0.093
20	0.076	0.052	0.060	0.054	0.060	0.102
19	0.078	0.049	0.052	0.050	0.052	0.104
18	0.079	0.045	0.043	0.046	0.043	0.090
17	0.069	0.041	0.035	0.041	0.035	0.088
16	0.065	0.037	0.028	0.035	0.028	0.075
15	0.060	0.032	0.022	0.030	0.022	0.061
14	0.054	0.028	0.017	0.025	0.017	0.048
13	0.047	0.023	0.012	0.021	0.012	0.035
12	0.040	0.019	0.009	0.017	0.009	0.025
11	0.033	0.015	0.006	0.013	0.006	0.017
10	0.026	0.012	0.004	0.010	0.004	0.011
9	0.020	0.009	0.003	0.007	0.003	0.007
8	0.015	0.007	0.002	0.005	0.002	0.004
7	0.011	0.005	0.001	0.003	0.009	0.002
6	0.007	0.003	0.000	0.002	0.000	0.001
5	0.004	0.002	0.000	0.001	0.000	0.000
4	0.002	0.001	0.000	0.001	0.000	0.000
3	0.001	0.000	0.000	0.000	0.000	0.000
2	0.000	0.000	0.000	0.000	0.000	0.000
1	0.000	0.000	0.000	0.000	0.000	0.000
Proportion of obsolescence peaks	0.62	0.61	0.63	0.60	0.63	0.63
Years of obsolescence peaks	15-23	17-28	20-28	18-28	20-28	16-22

Note: The data were calculated by Supplementary Table 12.

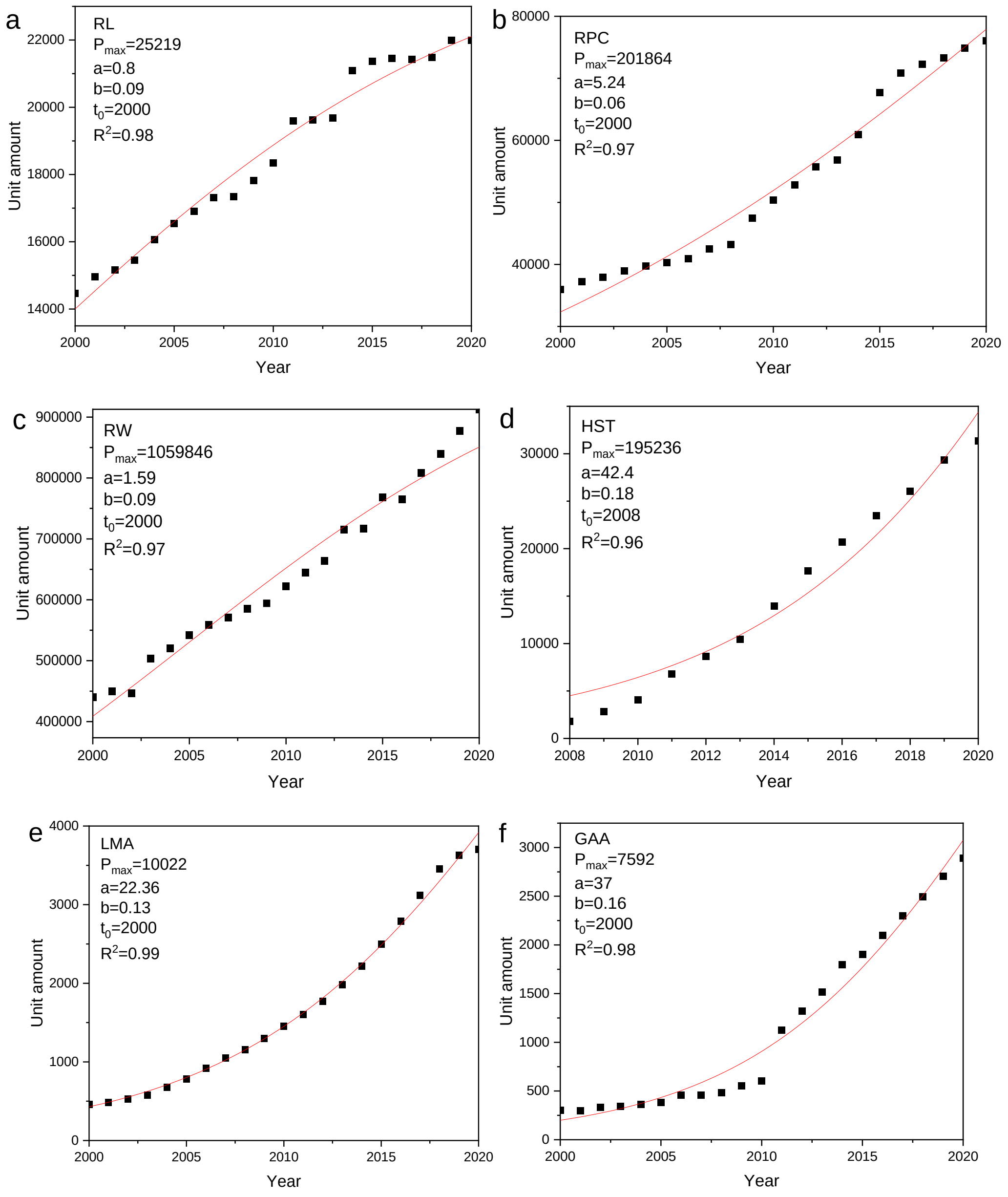

Supplementary Fig. 5 Data regression of possession amount of LPTV.

Supplementary Table 14 Market prices of Fe, Al, Nd, and Ti (US$/t)[24, 25, 26]

Resource	2017	2018	2019	2020	2021	Average	Error
Fe	442	416	432	432	436	432	±13
Al	2241	1962	1789	2028	2676	2139	±444
Nd	65831	64734	58307	96238	157523	88527	±49608
Ti	740	990	1256	1664	2189	1368	±725

a RPC

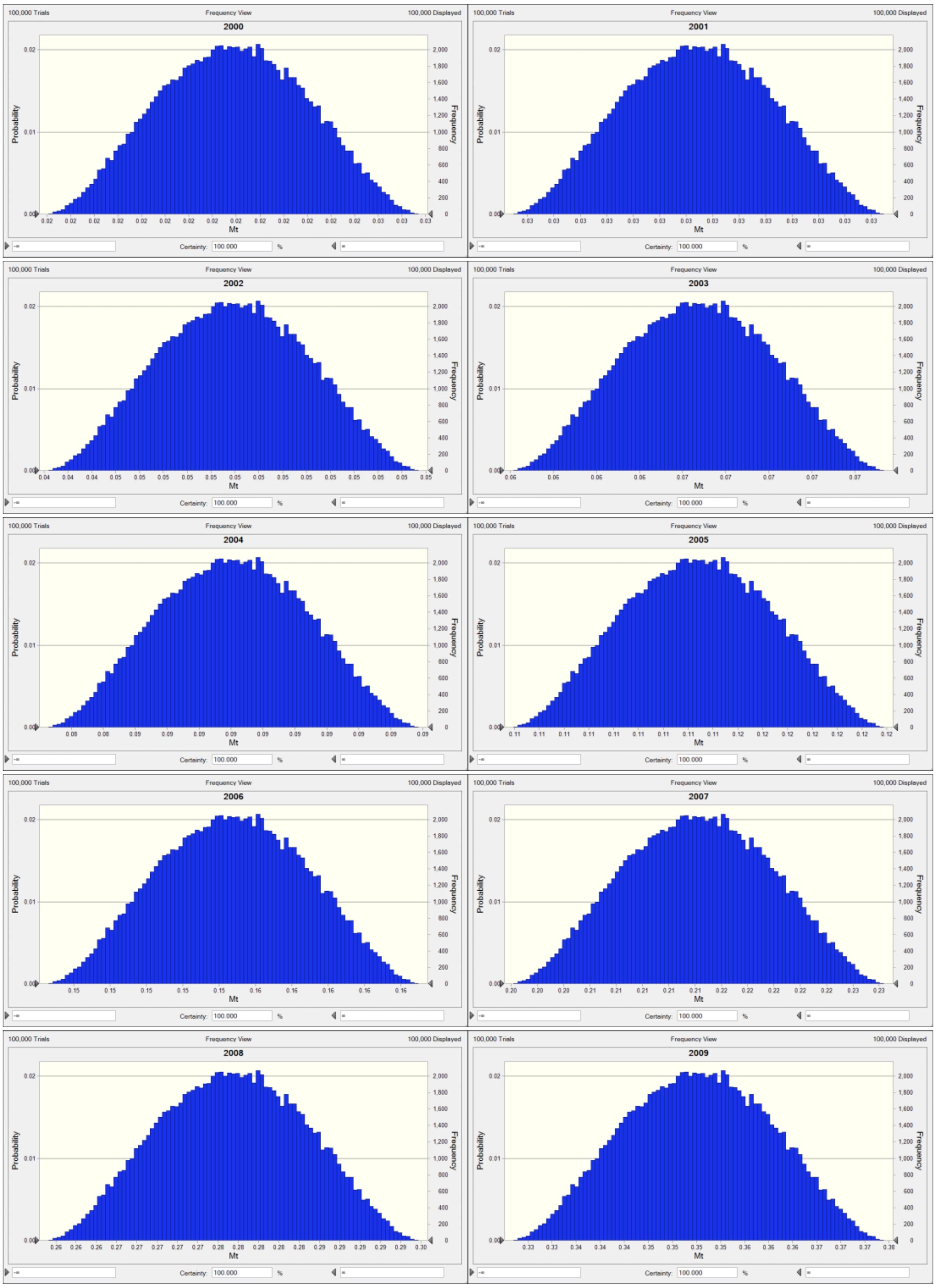

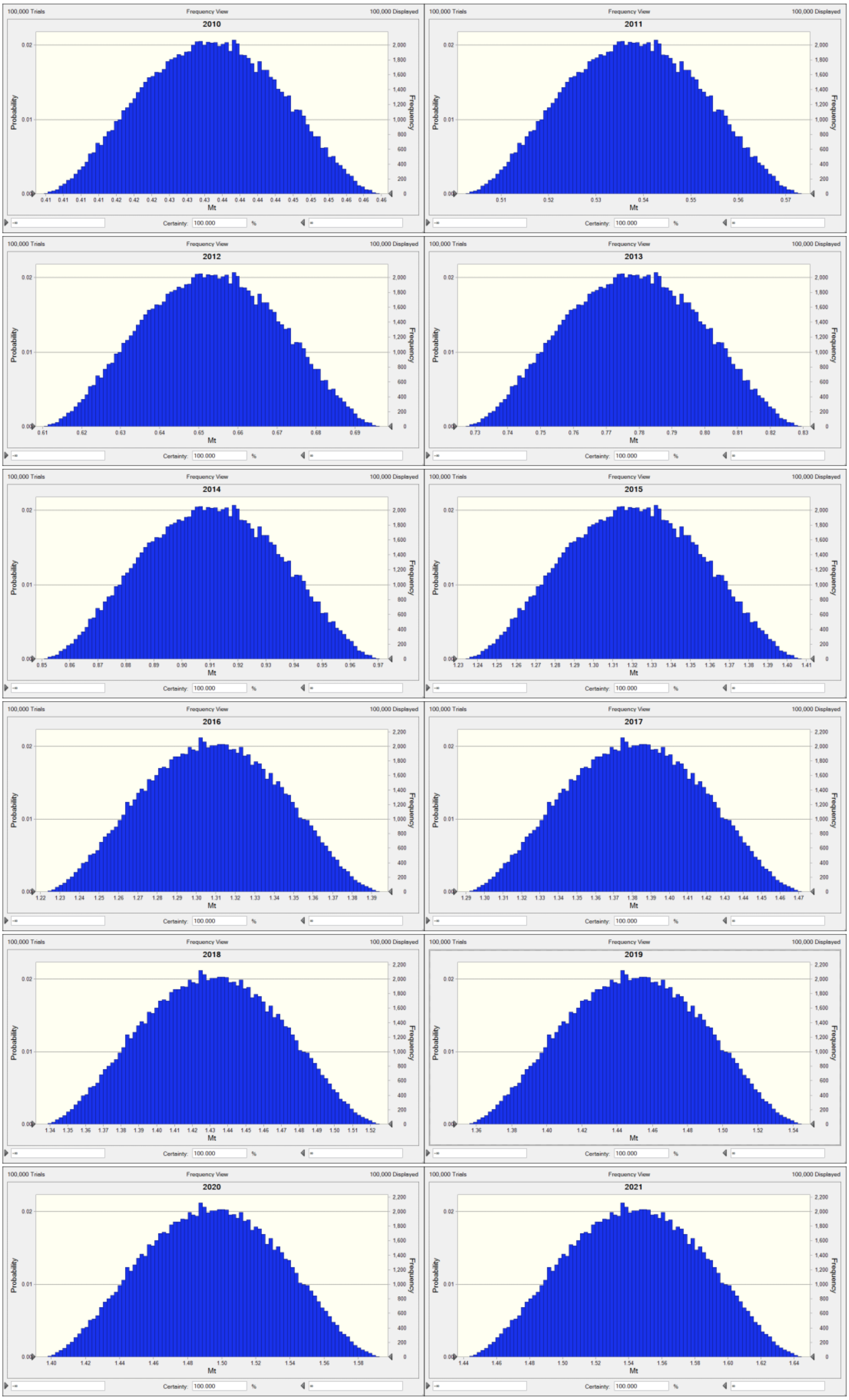
100,000 Trials
Frequency View
100,000 Displayed
2010
2011
2012
2013
2014
2015
2016
2017
2018
2019
2020
2021
Probability
Frequency
Mt
Certainty: 100.000 %

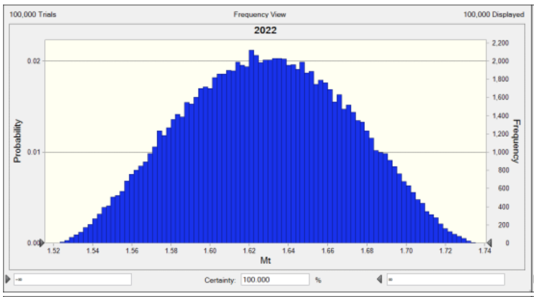
100,000 Trials
Frequency View
100,000 Displayed
2022
Probability
Frequency
1.52 1.54 1.56 1.58 1.60 1.62 1.64 1.66 1.68 1.70 1.72 1.74
Mt
Certainty: 100.000 %

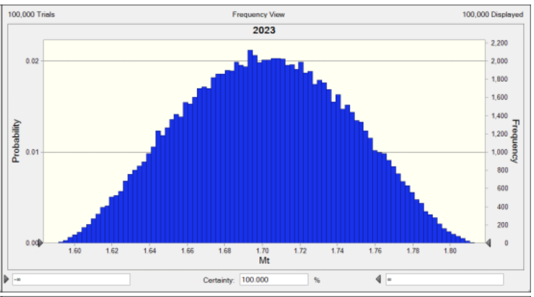
100,000 Trials
Frequency View
100,000 Displayed
2023
Probability
Frequency
1.60 1.62 1.64 1.66 1.68 1.70 1.72 1.74 1.76 1.78 1.80
Mt
Certainty: 100.000 %

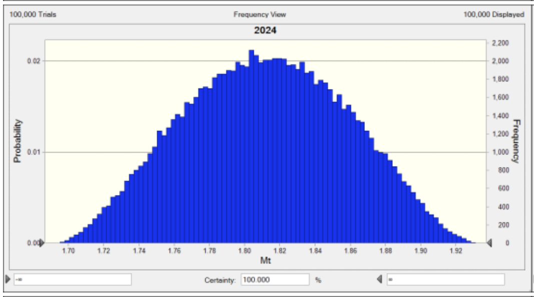
100,000 Trials
Frequency View
100,000 Displayed
2024
Probability
Frequency
1.70 1.72 1.74 1.76 1.78 1.80 1.82 1.84 1.86 1.88 1.90 1.92
Mt
Certainty: 100.000 %

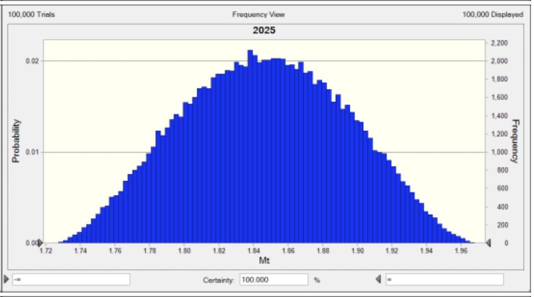
100,000 Trials
Frequency View
100,000 Displayed
2025
Probability
Frequency
1.72 1.74 1.76 1.78 1.80 1.82 1.84 1.86 1.88 1.90 1.92 1.94 1.96
Mt
Certainty: 100.000 %

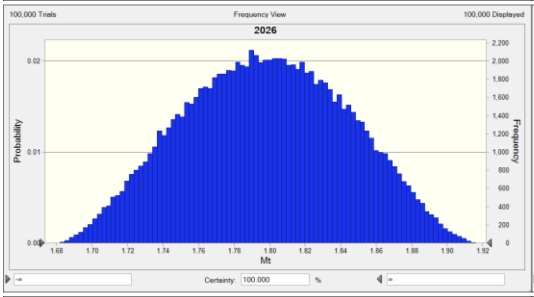
100,000 Trials
Frequency View
100,000 Displayed
2026
Probability
Frequency
1.68 1.70 1.72 1.74 1.76 1.78 1.80 1.82 1.84 1.86 1.88 1.90 1.92
Mt
Certainty: 100.000 %

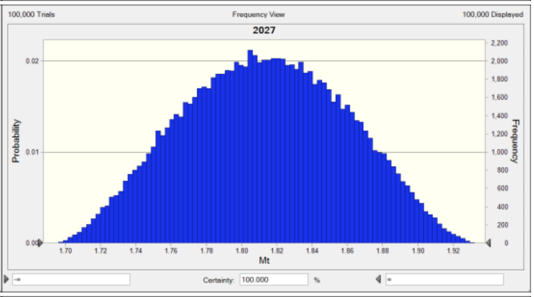
100,000 Trials
Frequency View
100,000 Displayed
2027
Probability
Frequency
1.70 1.72 1.74 1.76 1.78 1.80 1.82 1.84 1.86 1.88 1.90 1.92
Mt
Certainty: 100.000 %

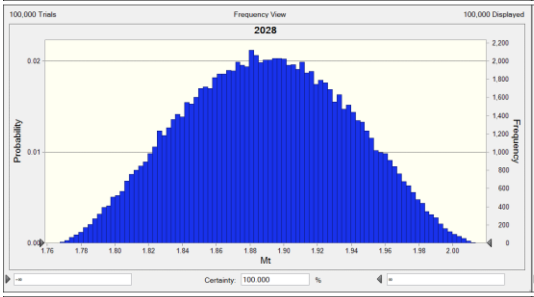
100,000 Trials
Frequency View
100,000 Displayed
2028
Probability
Frequency
1.76 1.78 1.80 1.82 1.84 1.86 1.88 1.90 1.92 1.94 1.96 1.98 2.00
Mt
Certainty: 100.000 %

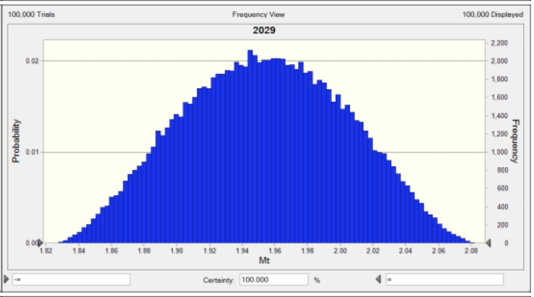
100,000 Trials
Frequency View
100,000 Displayed
2029
Probability
Frequency
1.82 1.84 1.86 1.88 1.90 1.92 1.94 1.96 1.98 2.00 2.02 2.04 2.06 2.08
Mt
Certainty: 100.000 %

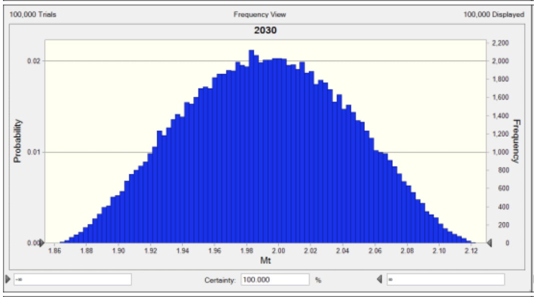
100,000 Trials
Frequency View
100,000 Displayed
2030
Probability
Frequency
1.86 1.88 1.90 1.92 1.94 1.96 1.98 2.00 2.02 2.04 2.06 2.08 2.10 2.12
Mt
Certainty: 100.000 %

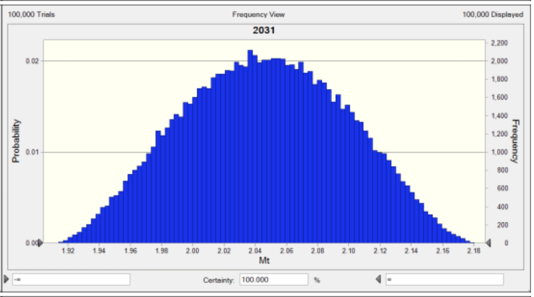
100,000 Trials
Frequency View
100,000 Displayed
2031
Probability
Frequency
1.92 1.94 1.96 1.98 2.00 2.02 2.04 2.06 2.08 2.10 2.12 2.14 2.16 2.18
Mt
Certainty: 100.000 %

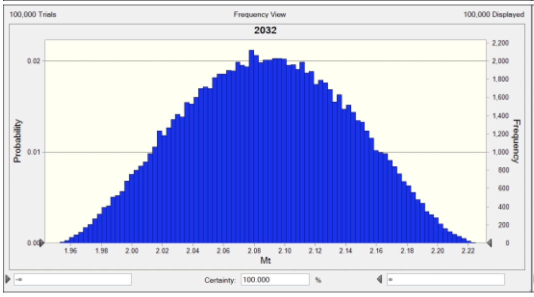
100,000 Trials
Frequency View
100,000 Displayed
2032
Probability
Frequency
1.96 1.98 2.00 2.02 2.04 2.06 2.08 2.10 2.12 2.14 2.16 2.18 2.20 2.22
Mt
Certainty: 100.000 %

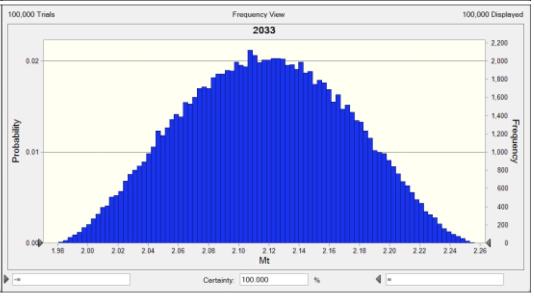
100,000 Trials
Frequency View
100,000 Displayed
2033
Probability
Frequency
1.98 2.00 2.02 2.04 2.06 2.08 2.10 2.12 2.14 2.16 2.18 2.20 2.22 2.24 2.26
Mt
Certainty: 100.000 %

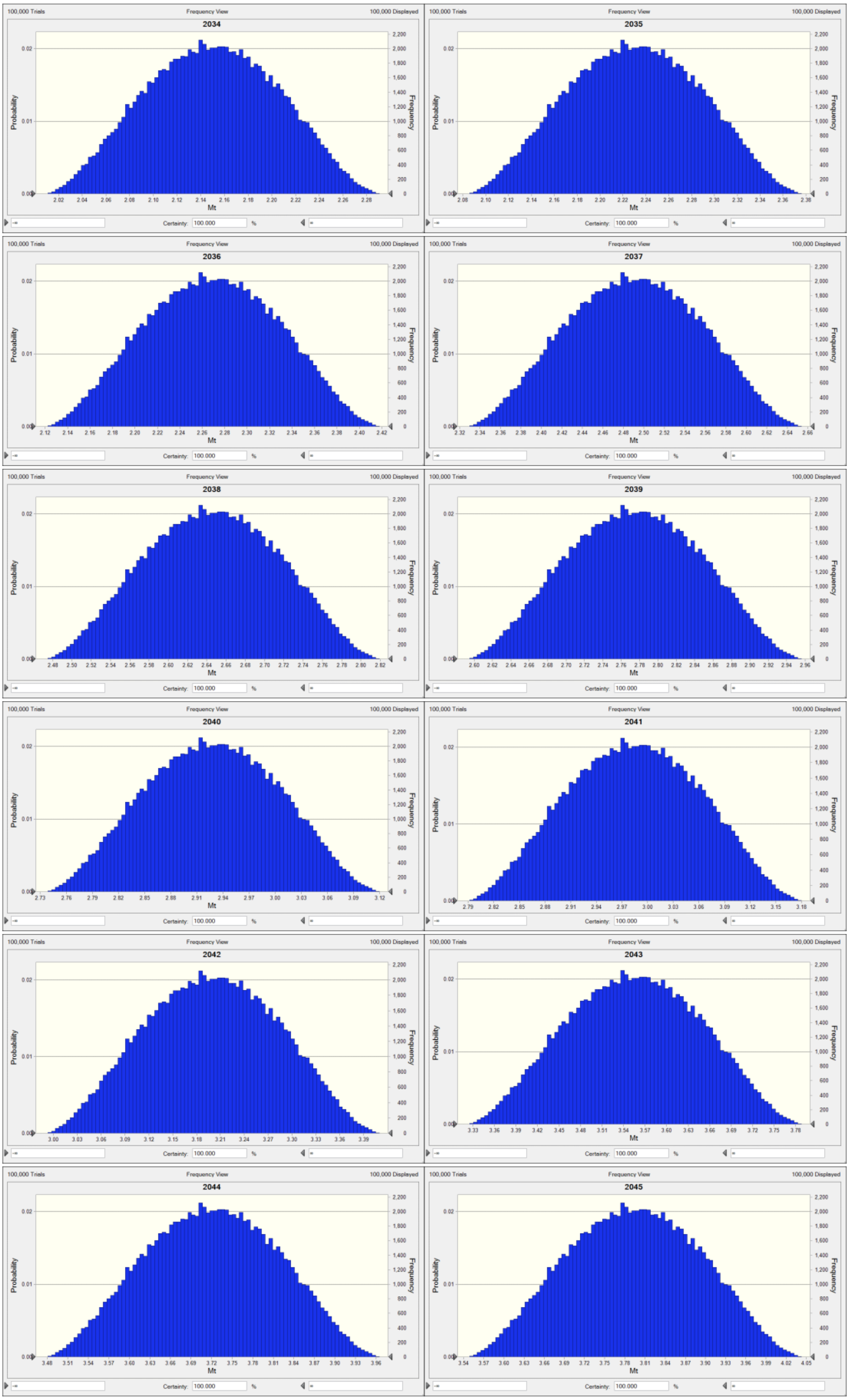
100,000 Trials
Frequency View
100,000 Displayed
2034
2035
2036
2037
2038
2039
2040
2041
2042
2043
2044
2045
Probability
Frequency
Mt
Certainty: 100.000 %

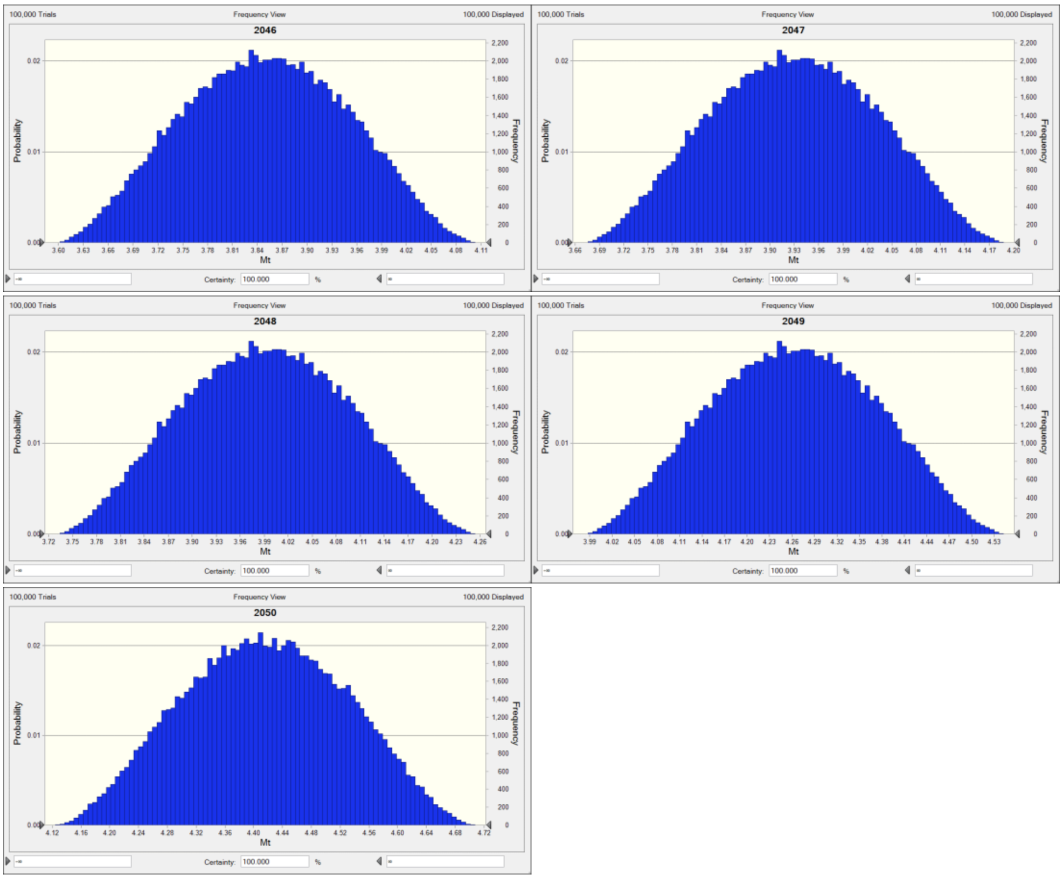
100,000 Trials
Frequency View
100,000 Displayed
2046
2047
2048
2049
2050
Probability
Frequency
Mt
Certainty: 100.000 %

b RL

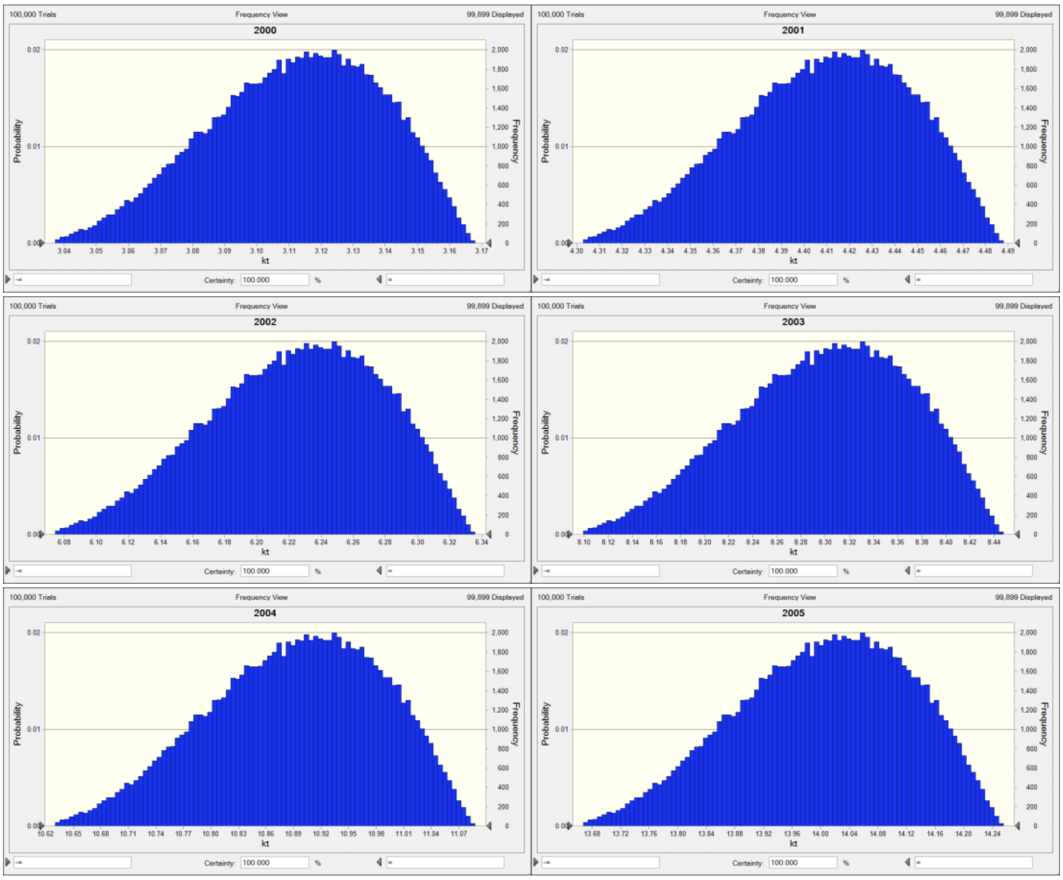
100,000 Trials
Frequency View
99,899 Displayed
2000
2001
2002
2003
2004
2005
Probability
Frequency
kt
Certainty: 100.000 %

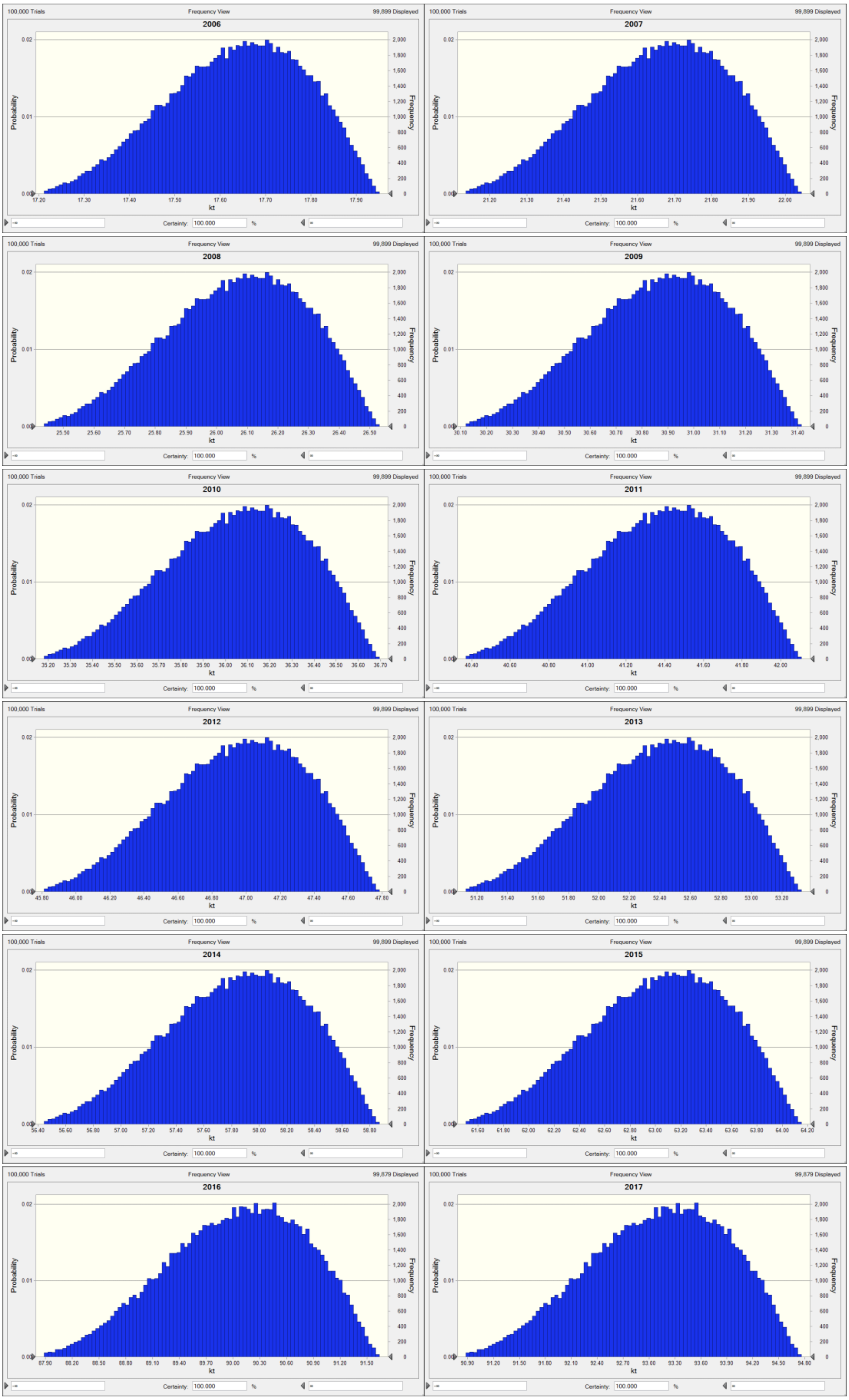
100,000 Trials
Frequency View
99,899 Displayed
2006
2007
2008
2009
2010
2011
2012
2013
2014
2015
2016
2017
99,879 Displayed
Probability
Frequency
kt
Certainty: 100.000 %

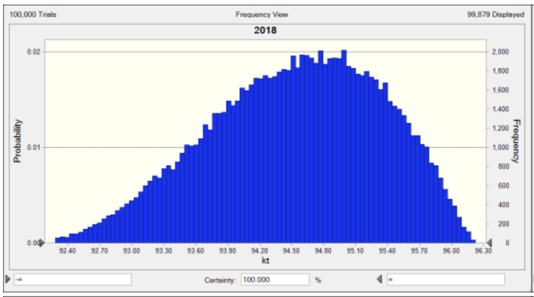
100,000 Trials
Frequency View
99,879 Displayed
2018
Probability
Frequency
kt
Certainty: 100.000 %

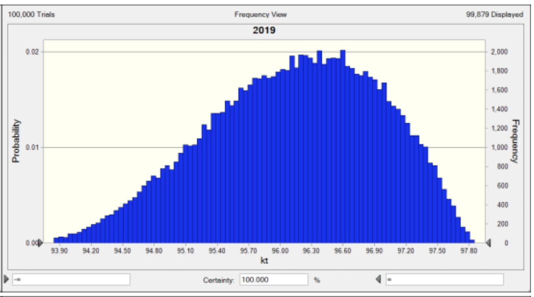
100,000 Trials
Frequency View
99,879 Displayed
2019
Probability
Frequency
kt
Certainty: 100.000 %

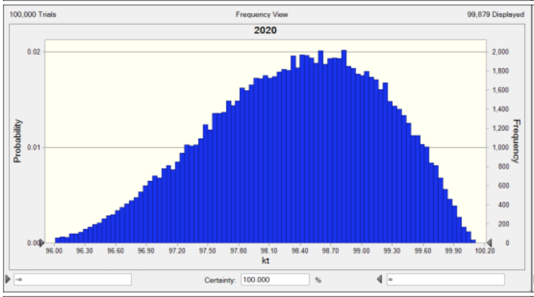
100,000 Trials
Frequency View
99,879 Displayed
2020
Probability
Frequency
kt
Certainty: 100.000 %

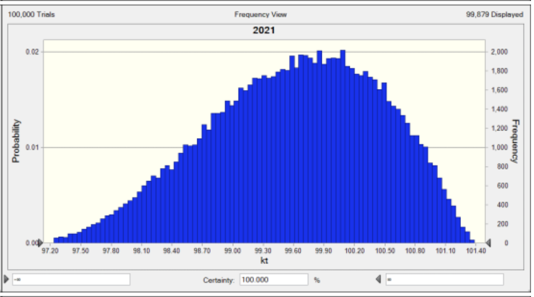
100,000 Trials
Frequency View
99,879 Displayed
2021
Probability
Frequency
kt
Certainty: 100.000 %

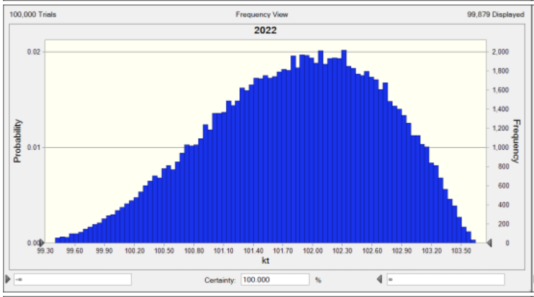
100,000 Trials
Frequency View
99,879 Displayed
2022
Probability
Frequency
kt
Certainty: 100.000 %

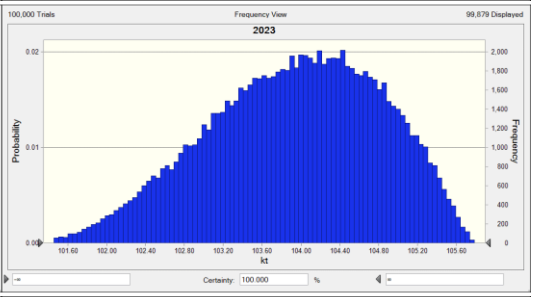
100,000 Trials
Frequency View
99,879 Displayed
2023
Probability
Frequency
kt
Certainty: 100.000 %

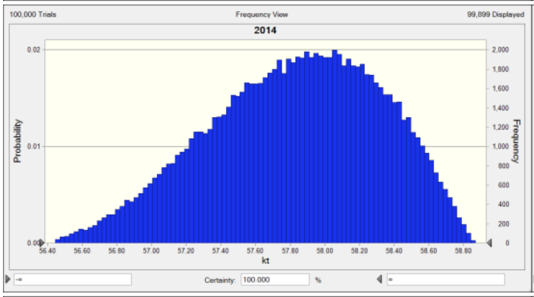
100,000 Trials
Frequency View
99,899 Displayed
2014
Probability
Frequency
kt
Certainty: 100.000 %

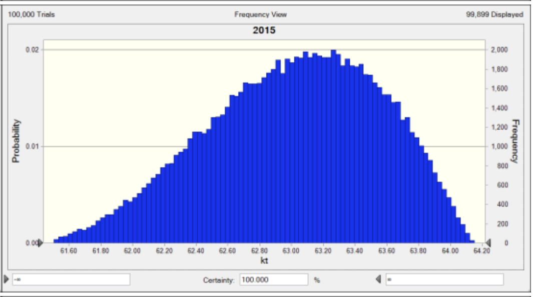
100,000 Trials
Frequency View
99,899 Displayed
2015
Probability
Frequency
kt
Certainty: 100.000 %

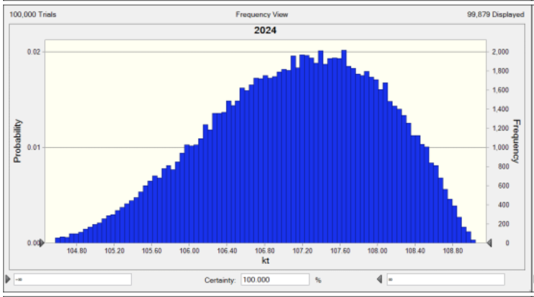
100,000 Trials
Frequency View
99,879 Displayed
2024
Probability
Frequency
kt
Certainty: 100.000 %

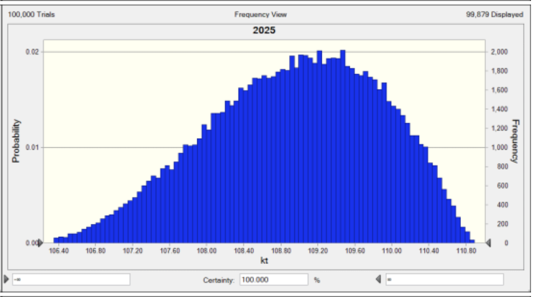
100,000 Trials
Frequency View
99,879 Displayed
2025
Probability
Frequency
kt
Certainty: 100.000 %

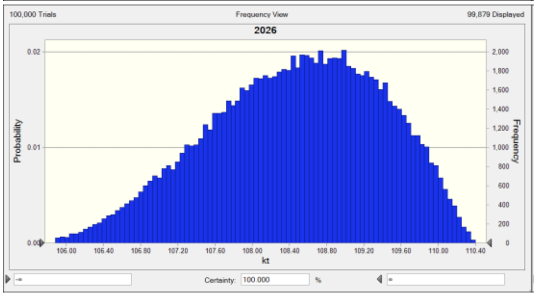
100,000 Trials
Frequency View
99,879 Displayed
2026
Probability
Frequency
kt
Certainty: 100.000 %

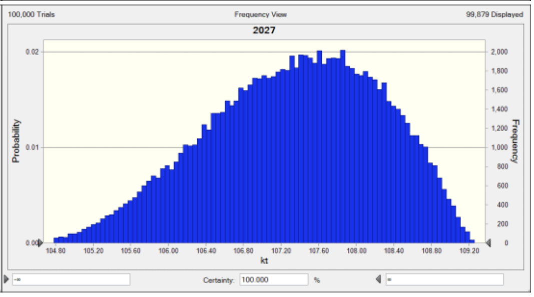
100,000 Trials
Frequency View
99,879 Displayed
2027
Probability
Frequency
kt
Certainty: 100.000 %

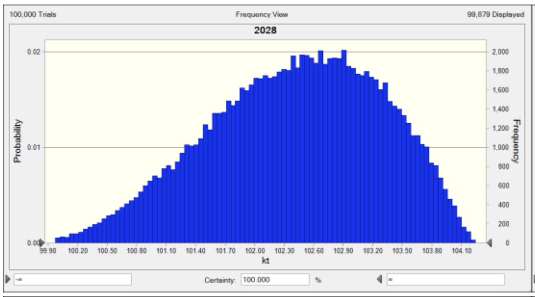
100,000 Trials
Frequency View
99,879 Displayed
2028
Probability
Frequency
kt
Certainty: 100.000 %

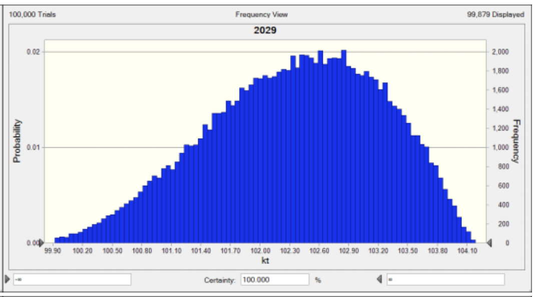
100,000 Trials
Frequency View
99,879 Displayed
2029
Probability
Frequency
kt
Certainty: 100.000 %

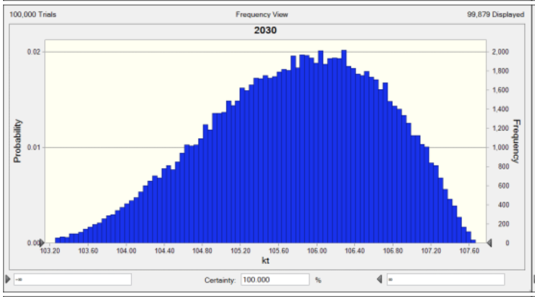
100,000 Trials
Frequency View
99,879 Displayed
2030
Probability
Frequency
kt
Certainty: 100.000 %

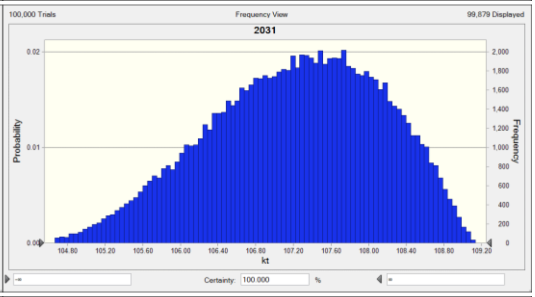
100,000 Trials
Frequency View
99,879 Displayed
2031
Probability
Frequency
kt
Certainty: 100.000 %

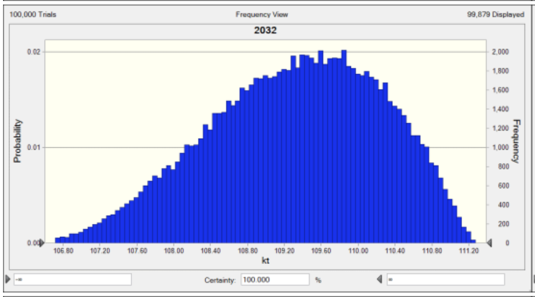
100,000 Trials
Frequency View
99,879 Displayed
2032
Probability
Frequency
kt
Certainty: 100.000 %

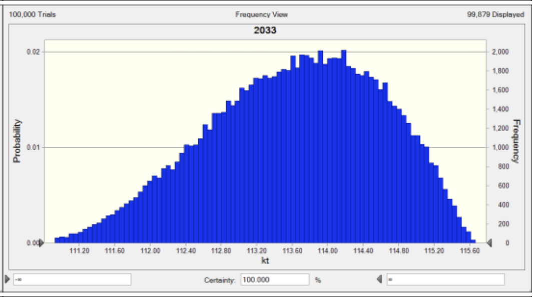
100,000 Trials
Frequency View
99,879 Displayed
2033
Probability
Frequency
kt
Certainty: 100.000 %

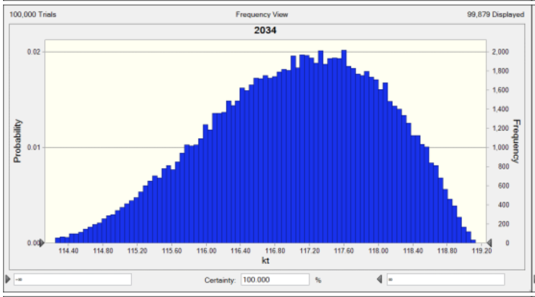
100,000 Trials
Frequency View
99,879 Displayed
2034
Probability
Frequency
kt
Certainty: 100.000 %

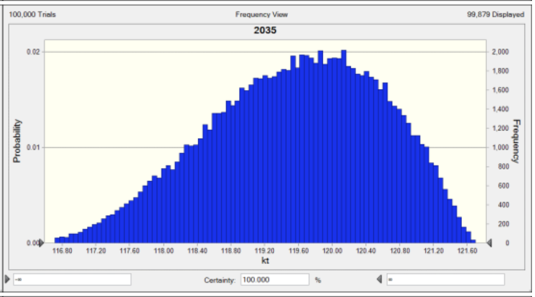
100,000 Trials
Frequency View
99,879 Displayed
2035
Probability
Frequency
kt
Certainty: 100.000 %

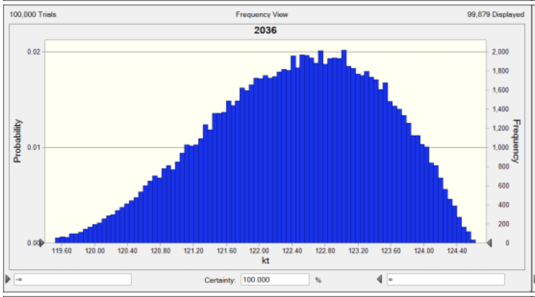
100,000 Trials
Frequency View
99,879 Displayed
2036
Probability
Frequency
kt
Certainty: 100.000 %

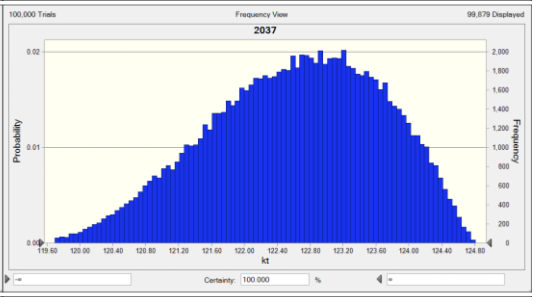
100,000 Trials
Frequency View
99,879 Displayed
2037
Probability
Frequency
kt
Certainty: 100.000 %

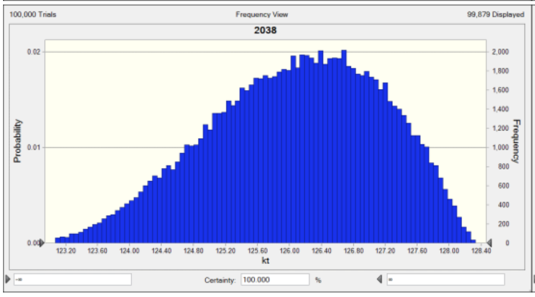
100,000 Trials
Frequency View
99,879 Displayed
2038
Probability
Frequency
kt
Certainty: 100.000 %

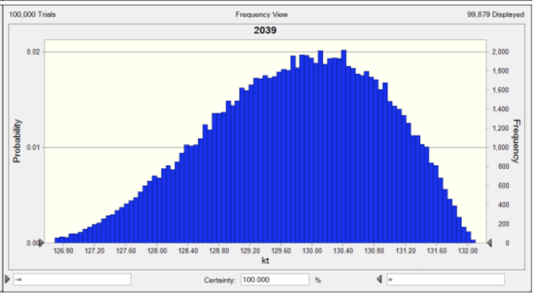
100,000 Trials
Frequency View
99,879 Displayed
2039
Probability
Frequency
kt
Certainty: 100.000 %

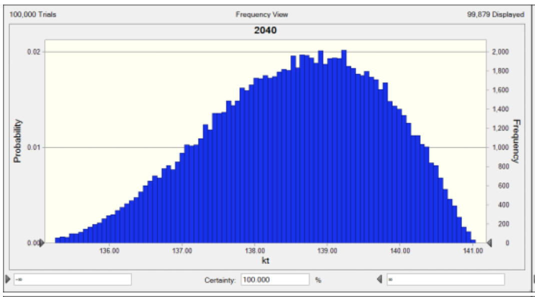
100,000 Trials
Frequency View
99,879 Displayed
2040
Probability
Frequency
136.00 137.00 138.00 139.00 140.00 141.00
kt
Certainty: 100.000 %

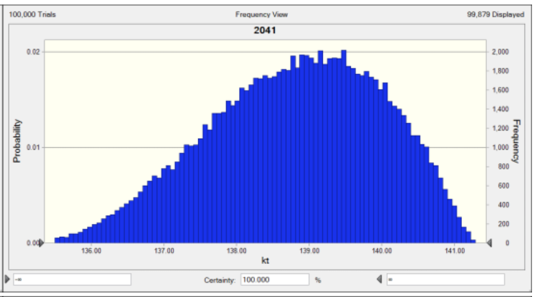
100,000 Trials
Frequency View
99,879 Displayed
2041
Probability
Frequency
136.00 137.00 138.00 139.00 140.00 141.00
kt
Certainty: 100.000 %

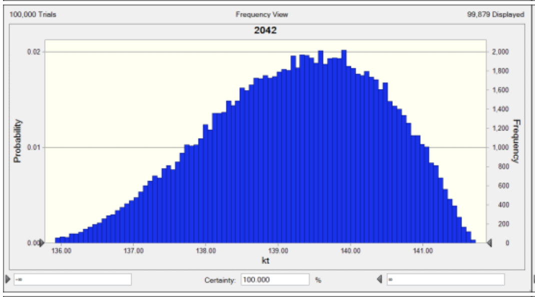
100,000 Trials
Frequency View
99,879 Displayed
2042
Probability
Frequency
136.00 137.00 138.00 139.00 140.00 141.00
kt
Certainty: 100.000 %

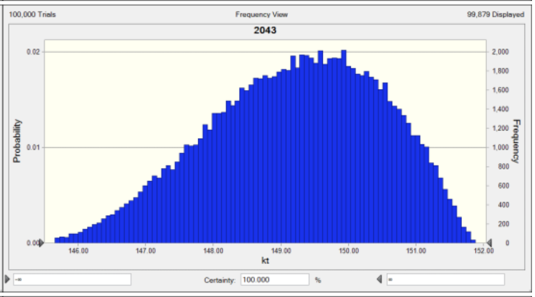
100,000 Trials
Frequency View
99,879 Displayed
2043
Probability
Frequency
146.00 147.00 148.00 149.00 150.00 151.00 152.00
kt
Certainty: 100.000 %

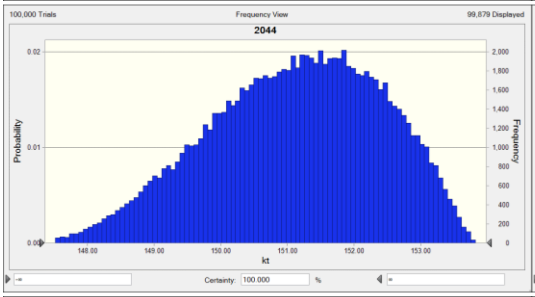
100,000 Trials
Frequency View
99,879 Displayed
2044
Probability
Frequency
148.00 149.00 150.00 151.00 152.00 153.00
kt
Certainty: 100.000 %

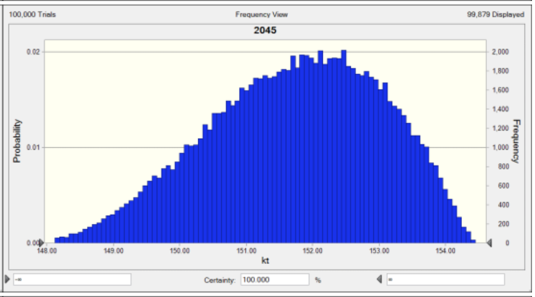
100,000 Trials
Frequency View
99,879 Displayed
2045
Probability
Frequency
148.00 149.00 150.00 151.00 152.00 153.00 154.00
kt
Certainty: 100.000 %

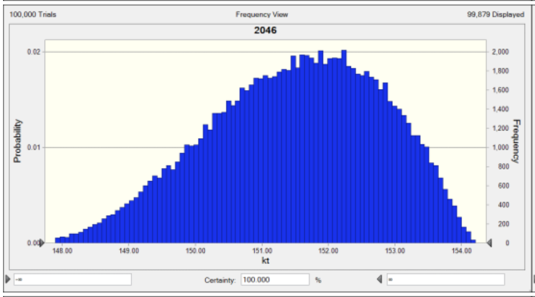
100,000 Trials
Frequency View
99,879 Displayed
2046
Probability
Frequency
148.00 149.00 150.00 151.00 152.00 153.00 154.00
kt
Certainty: 100.000 %

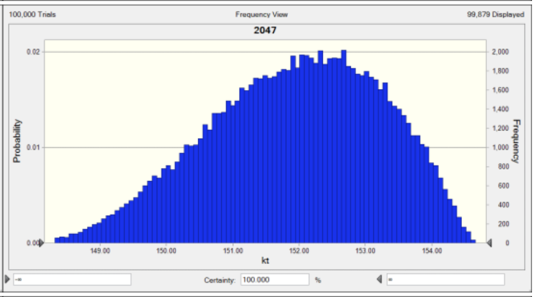
100,000 Trials
Frequency View
99,879 Displayed
2047
Probability
Frequency
149.00 150.00 151.00 152.00 153.00 154.00
kt
Certainty: 100.000 %

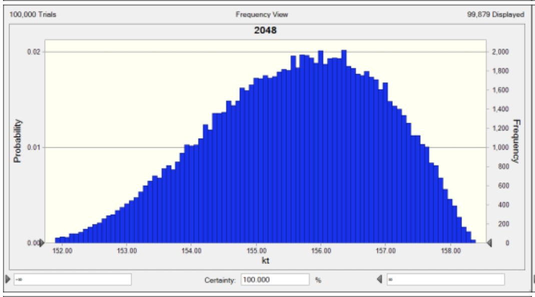
100,000 Trials
Frequency View
99,879 Displayed
2048
Probability
Frequency
152.00 153.00 154.00 155.00 156.00 157.00 158.00
kt
Certainty: 100.000 %

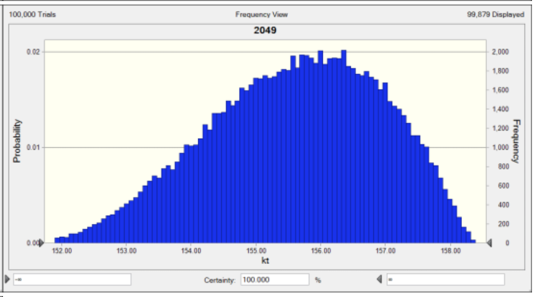
100,000 Trials
Frequency View
99,879 Displayed
2049
Probability
Frequency
152.00 153.00 154.00 155.00 156.00 157.00 158.00
kt
Certainty: 100.000 %

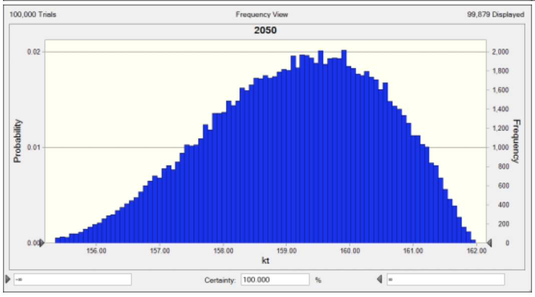
100,000 Trials
Frequency View
99,879 Displayed
2050
Probability
Frequency
156.00 157.00 158.00 159.00 160.00 161.00 162.00
kt
Certainty: 100.000 %

c RW

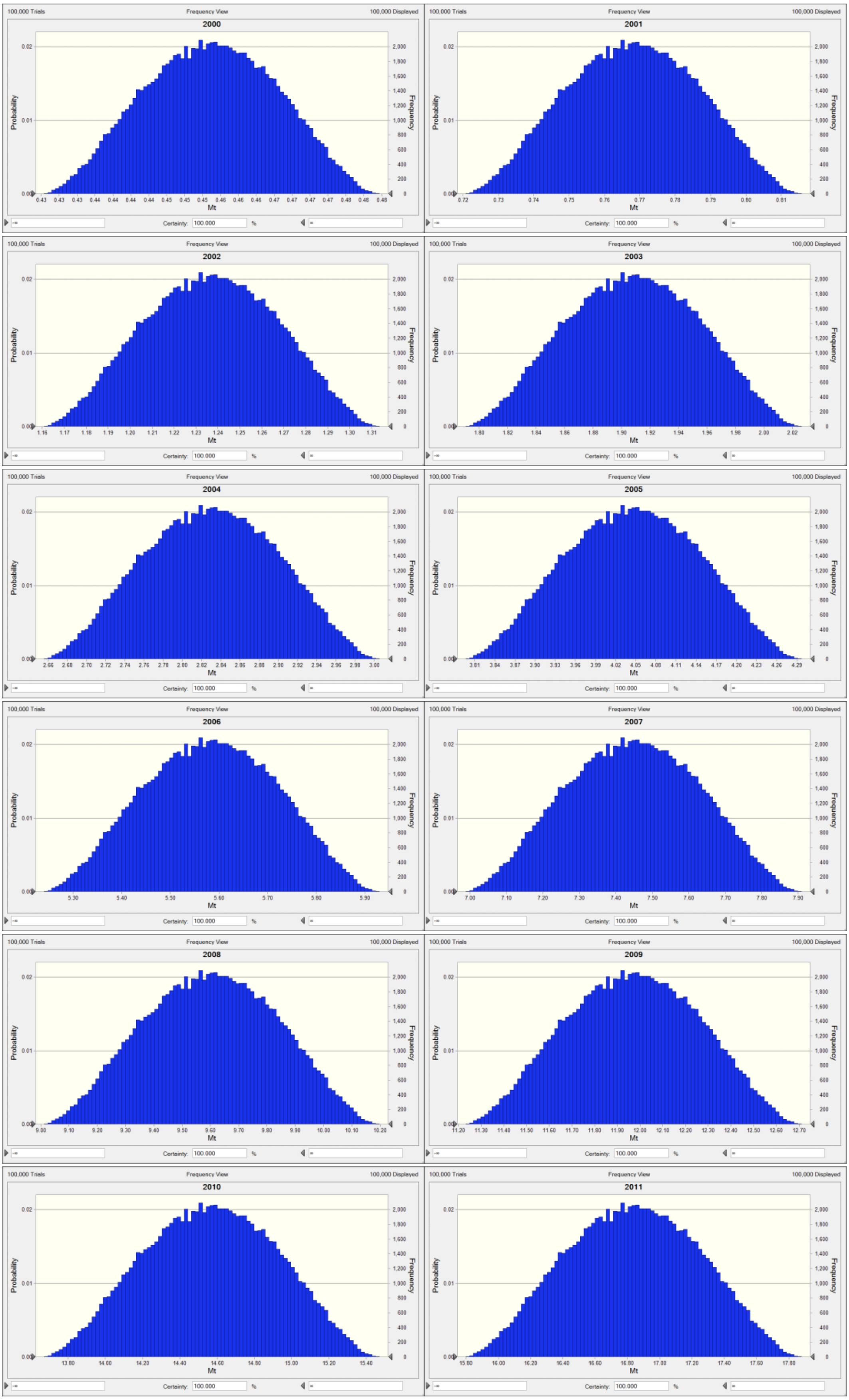
100,000 Trials
Frequency View
100,000 Displayed
2000
2001
2002
2003
2004
2005
2006
2007
2008
2009
2010
2011
Probability
Frequency
Mt
Certainty: 100.000 %

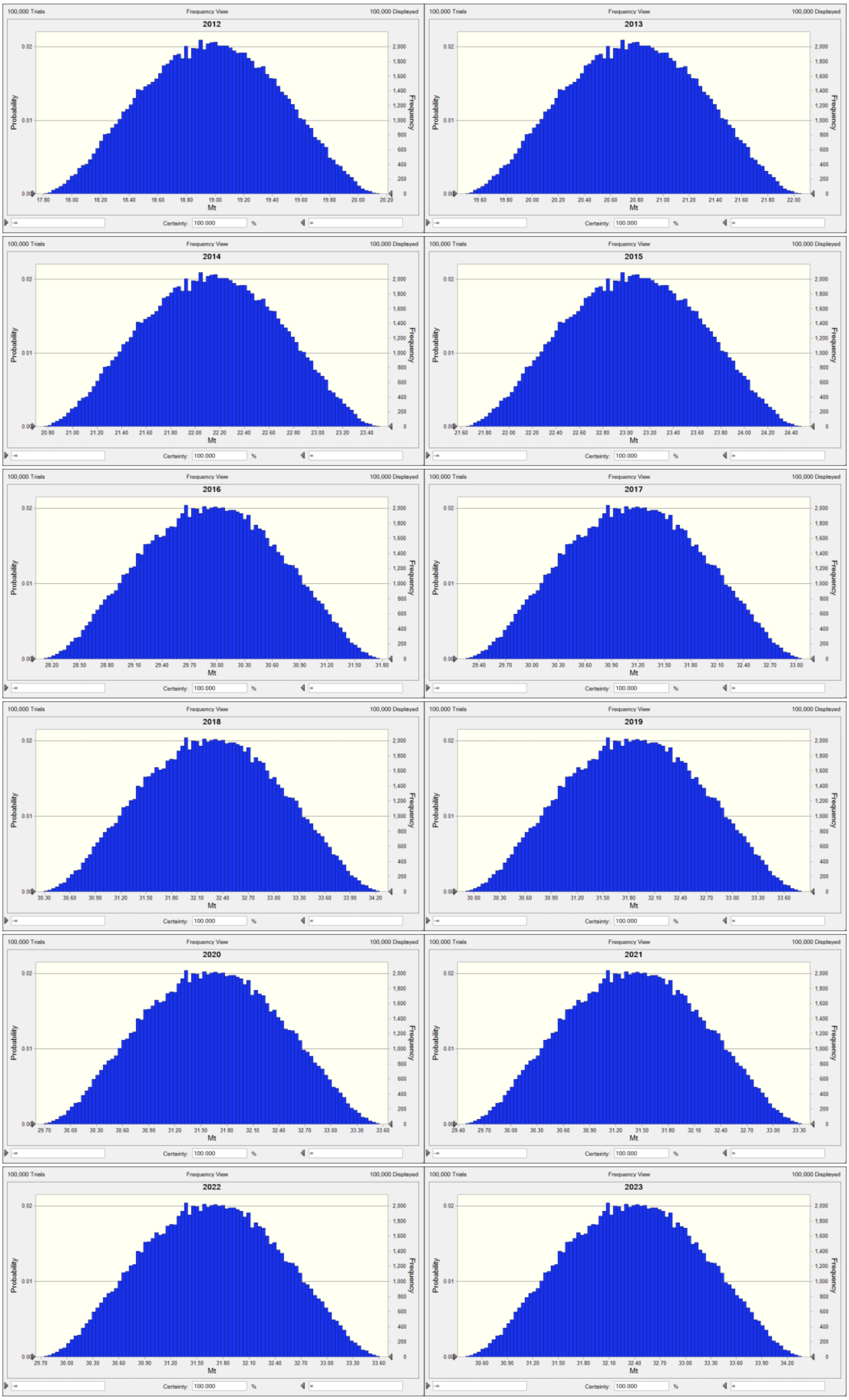
100,000 Trials
Frequency View
100,000 Displayed
2012
2013
2014
2015
2016
2017
2018
2019
2020
2021
2022
2023
Probability
Frequency
Mt
Certainty: 100.000 %

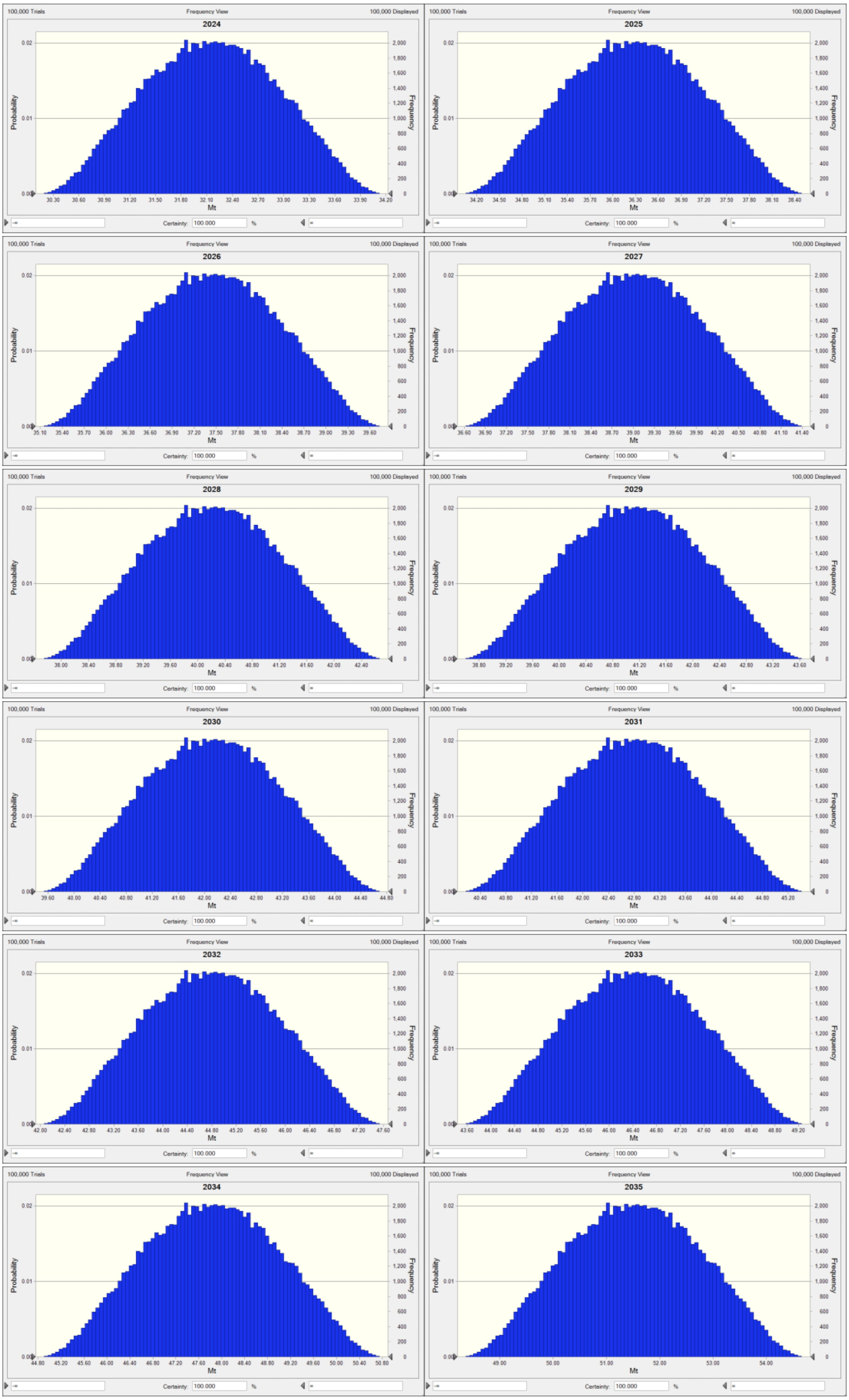
100,000 Trials
Frequency View
100,000 Displayed
2024
2025
2026
2027
2028
2029
2030
2031
2032
2033
2034
2035
Probability
Frequency
Mt
Certainty: 100.000 %

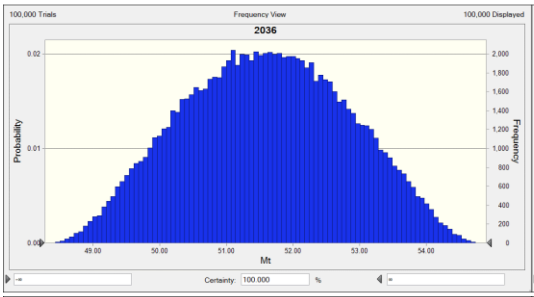
100,000 Trials
Frequency View
100,000 Displayed
2036
Probability
Frequency
Mt
Certainty: 100.000 %

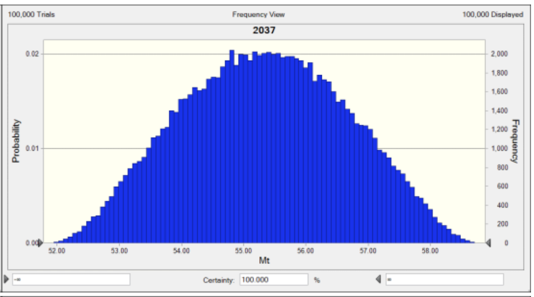
100,000 Trials
Frequency View
100,000 Displayed
2037
Probability
Frequency
Mt
Certainty: 100.000 %

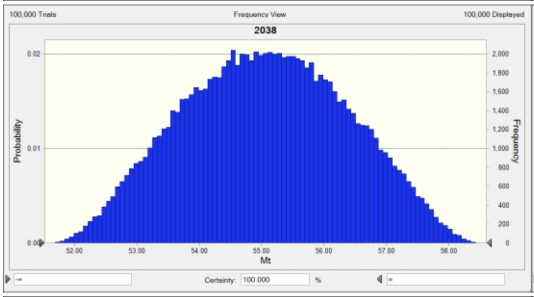
100,000 Trials
Frequency View
100,000 Displayed
2038
Probability
Frequency
Mt
Certainty: 100.000 %

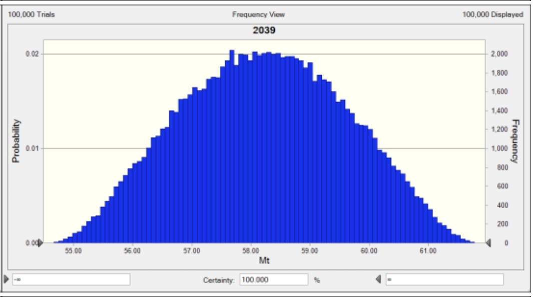
100,000 Trials
Frequency View
100,000 Displayed
2039
Probability
Frequency
Mt
Certainty: 100.000 %

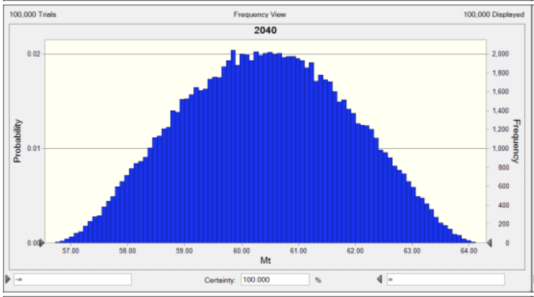
100,000 Trials
Frequency View
100,000 Displayed
2040
Probability
Frequency
Mt
Certainty: 100.000 %

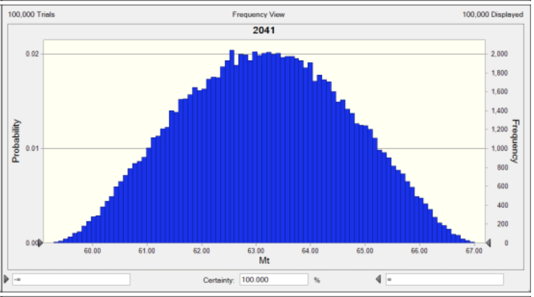
100,000 Trials
Frequency View
100,000 Displayed
2041
Probability
Frequency
Mt
Certainty: 100.000 %

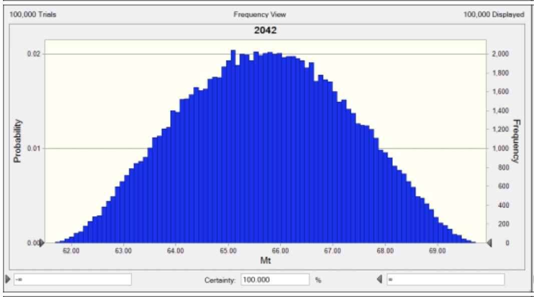
100,000 Trials
Frequency View
100,000 Displayed
2042
Probability
Frequency
Mt
Certainty: 100.000 %

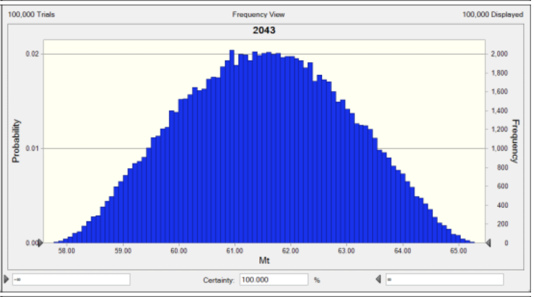
100,000 Trials
Frequency View
100,000 Displayed
2043
Probability
Frequency
Mt
Certainty: 100.000 %

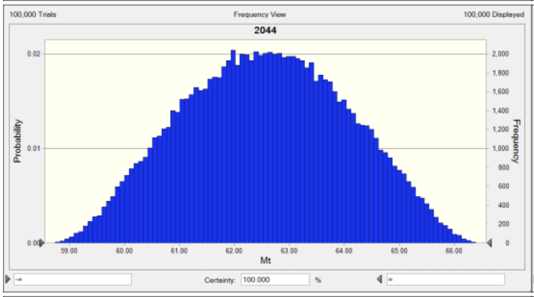
100,000 Trials
Frequency View
100,000 Displayed
2044
Probability
Frequency
Mt
Certainty: 100.000 %

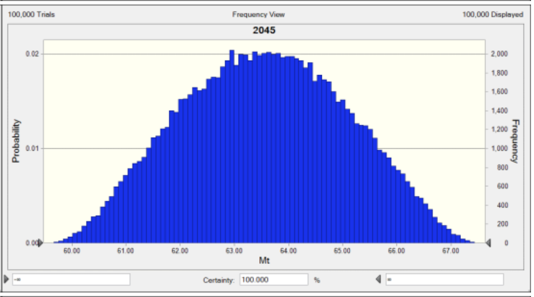
100,000 Trials
Frequency View
100,000 Displayed
2045
Probability
Frequency
Mt
Certainty: 100.000 %

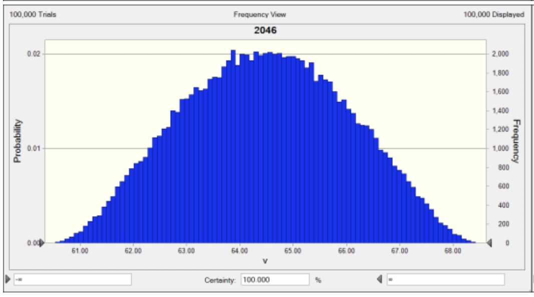
100,000 Trials
Frequency View
100,000 Displayed
2046
Probability
Frequency
v
Certainty: 100.000 %

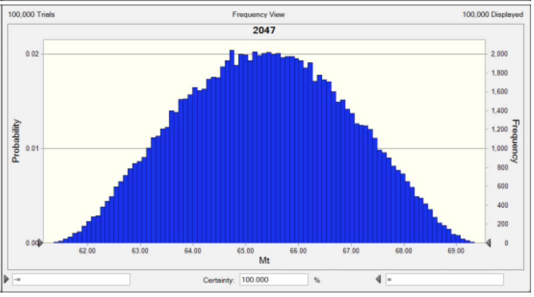
100,000 Trials
Frequency View
100,000 Displayed
2047
Probability
Frequency
Mt
Certainty: 100.000 %

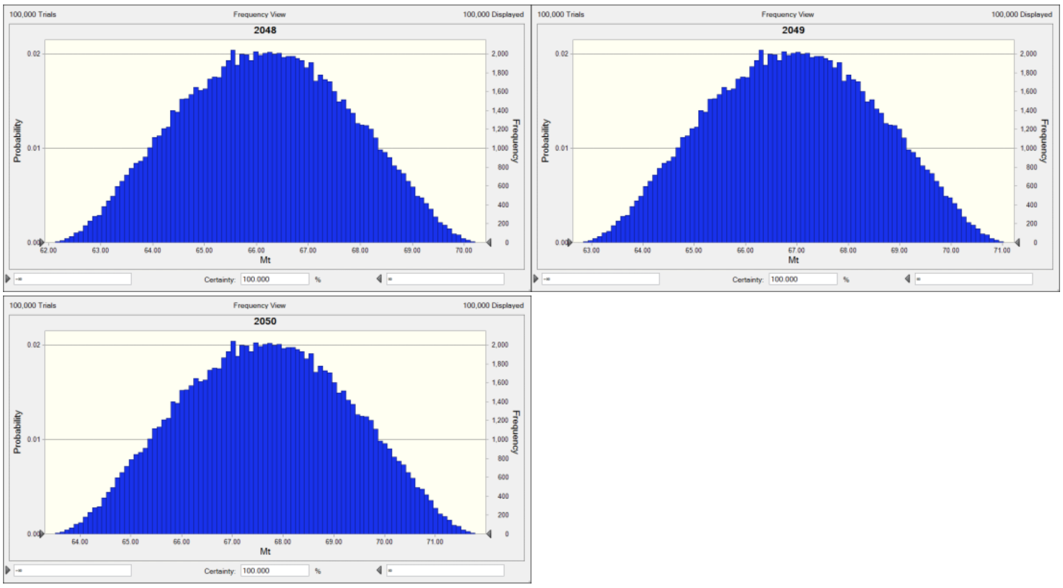
100,000 Trials
Frequency View
100,000 Displayed
2048
2049
2050
Probability
Frequency
Mt
Certainty: 100.000 %

d HST

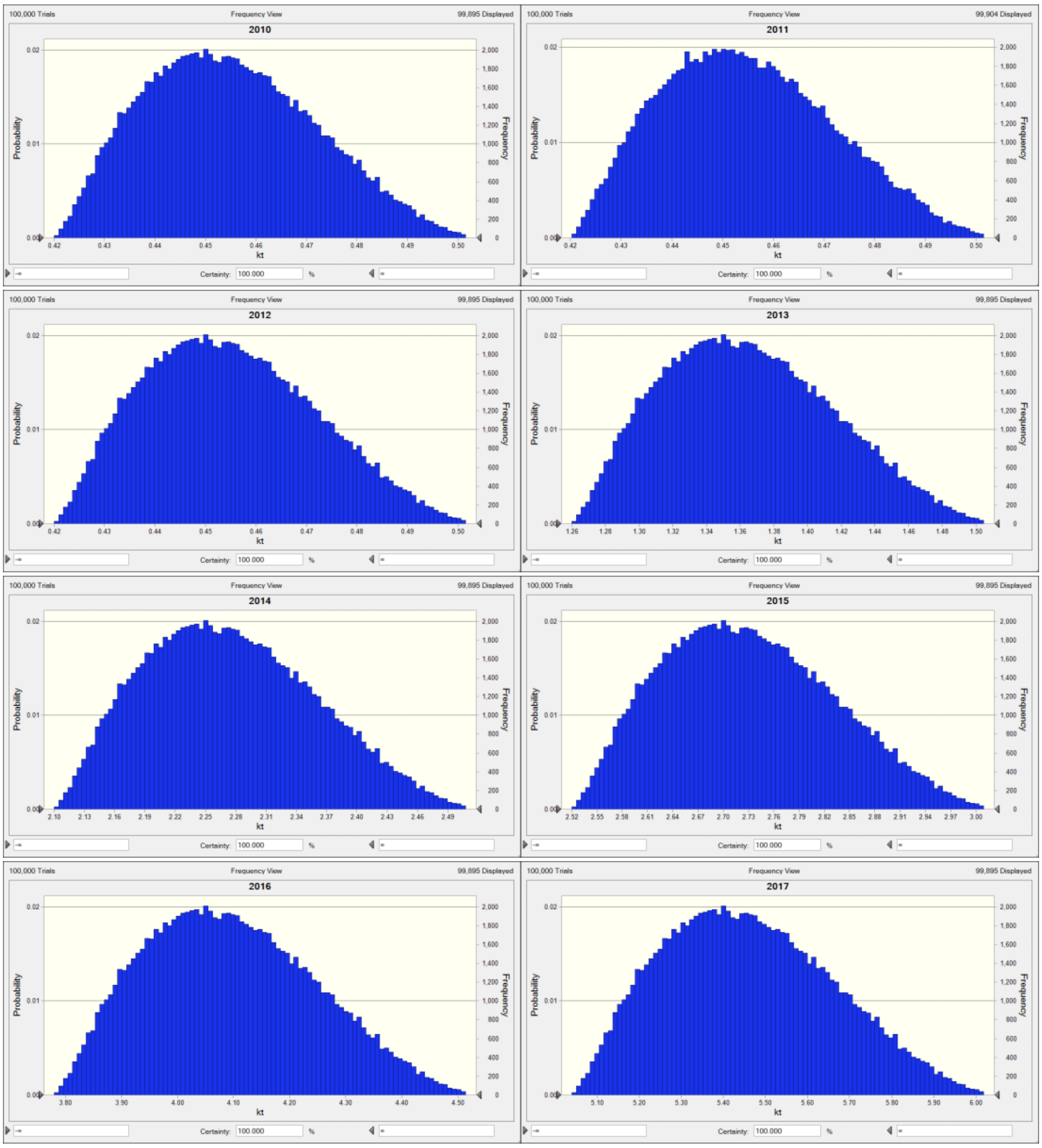
100,000 Trials
Frequency View
99,895 Displayed
99,904 Displayed
2010
2011
2012
2013
2014
2015
2016
2017
Probability
Frequency
kt
Certainty: 100.000 %

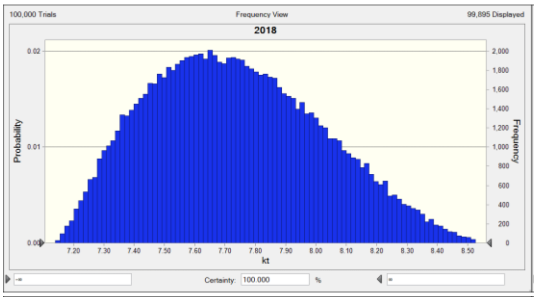
100,000 Trials
Frequency View
99,895 Displayed
2018
Probability
Frequency
kt
Certainty: 100.000 %

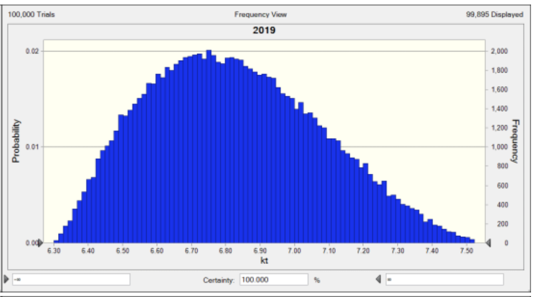
100,000 Trials
Frequency View
99,895 Displayed
2019
Probability
Frequency
kt
Certainty: 100.000 %

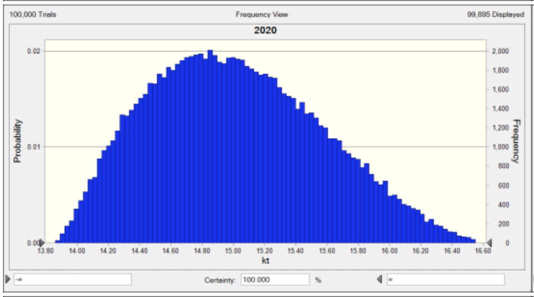
100,000 Trials
Frequency View
99,895 Displayed
2020
Probability
Frequency
kt
Certainty: 100.000 %

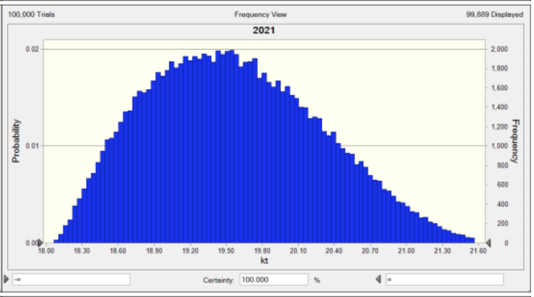
100,000 Trials
Frequency View
99,889 Displayed
2021
Probability
Frequency
kt
Certainty: 100.000 %

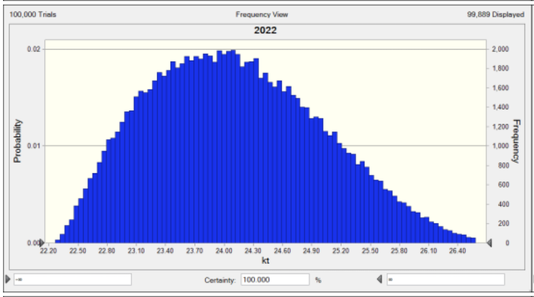
100,000 Trials
Frequency View
99,889 Displayed
2022
Probability
Frequency
kt
Certainty: 100.000 %

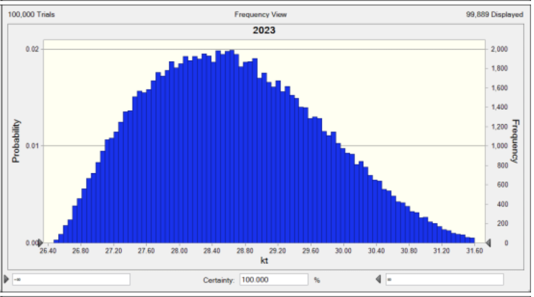
100,000 Trials
Frequency View
99,889 Displayed
2023
Probability
Frequency
kt
Certainty: 100.000 %

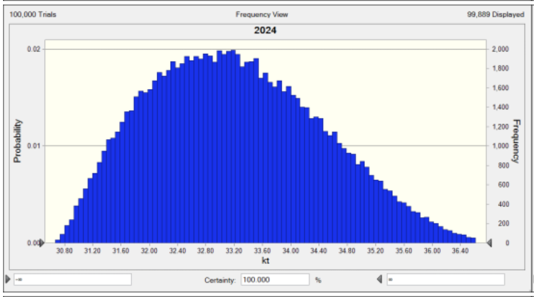
100,000 Trials
Frequency View
99,889 Displayed
2024
Probability
Frequency
kt
Certainty: 100.000 %

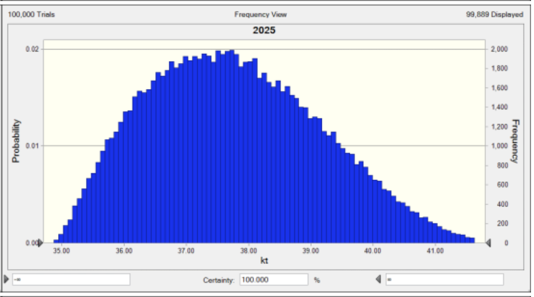
100,000 Trials
Frequency View
99,889 Displayed
2025
Probability
Frequency
kt
Certainty: 100.000 %

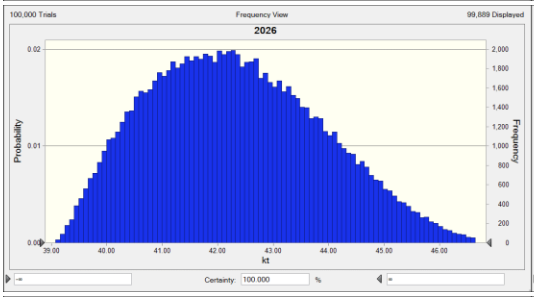
100,000 Trials
Frequency View
99,889 Displayed
2026
Probability
Frequency
kt
Certainty: 100.000 %

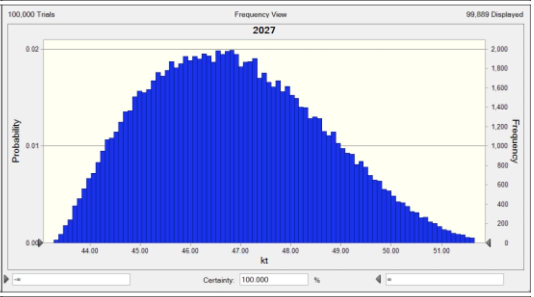
100,000 Trials
Frequency View
99,889 Displayed
2027
Probability
Frequency
kt
Certainty: 100.000 %

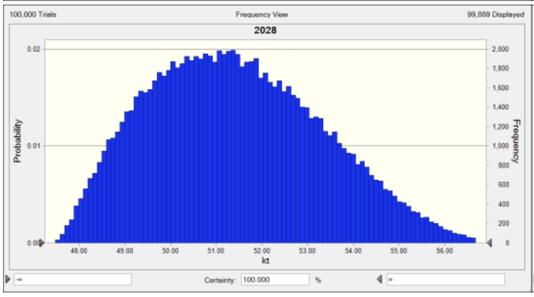
100,000 Trials
Frequency View
99,889 Displayed
2028
Probability
Frequency
kt
Certainty: 100.000 %

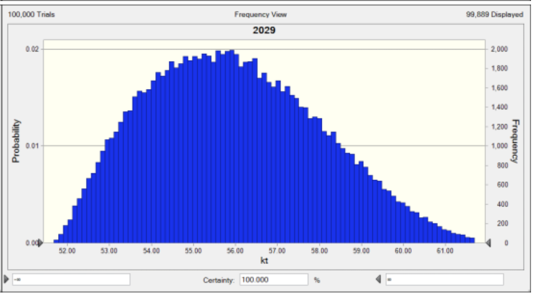
100,000 Trials
Frequency View
99,889 Displayed
2029
Probability
Frequency
kt
Certainty: 100.000 %

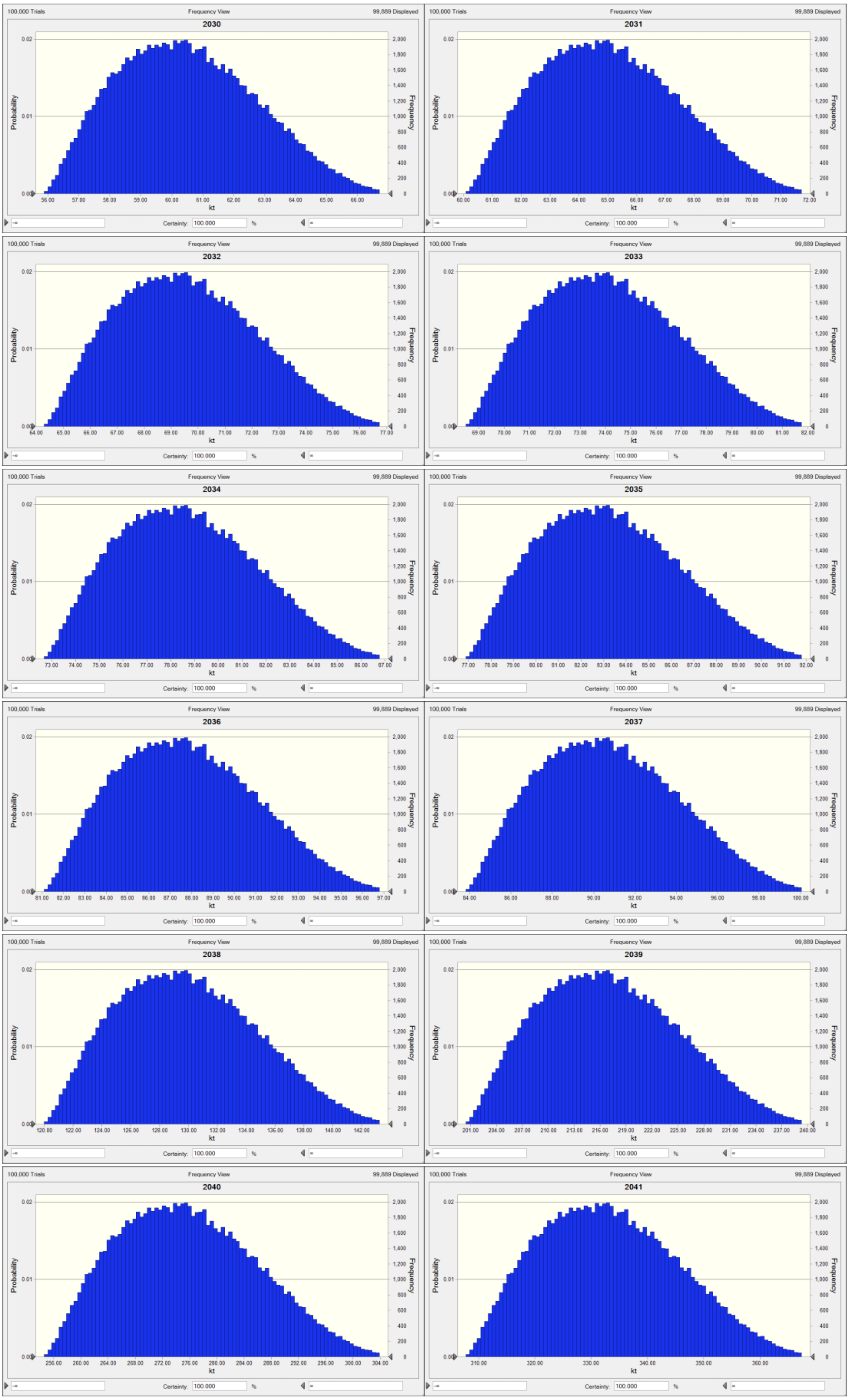
100,000 Trials
Frequency View
99,889 Displayed
2030
2031
2032
2033
2034
2035
2036
2037
2038
2039
2040
2041
Probability
Frequency
kt
Certainty: 100.000 %

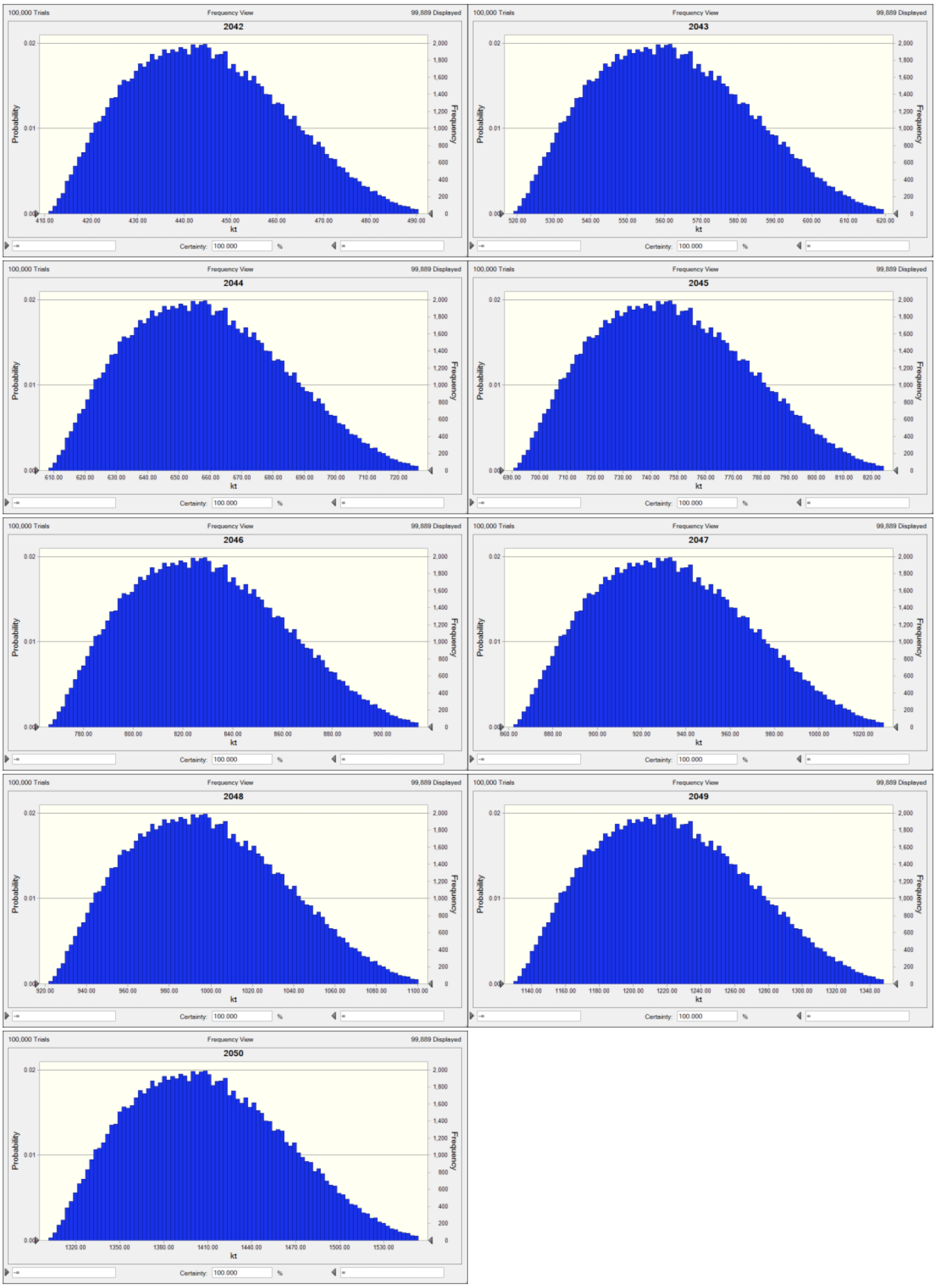
100,000 Trials
Frequency View
99,889 Displayed
2042
2043
2044
2045
2046
2047
2048
2049
2050
Probability
Frequency
kt
Certainty: 100.000 %

e LMA

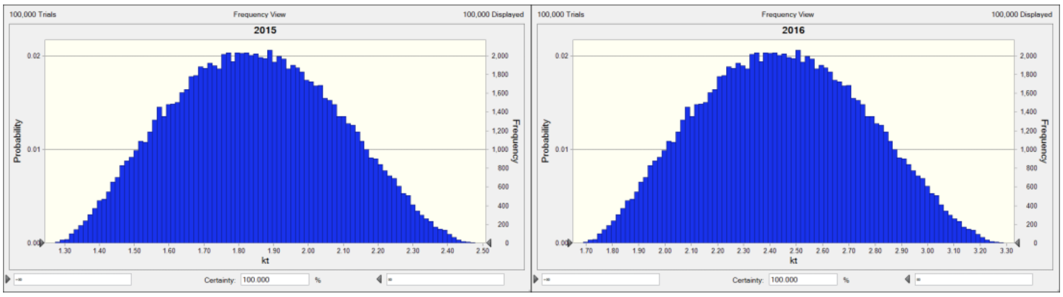
100,000 Trials
Frequency View
100,000 Displayed
2015
2016
Probability
Frequency
kt
Certainty: 100.000 %

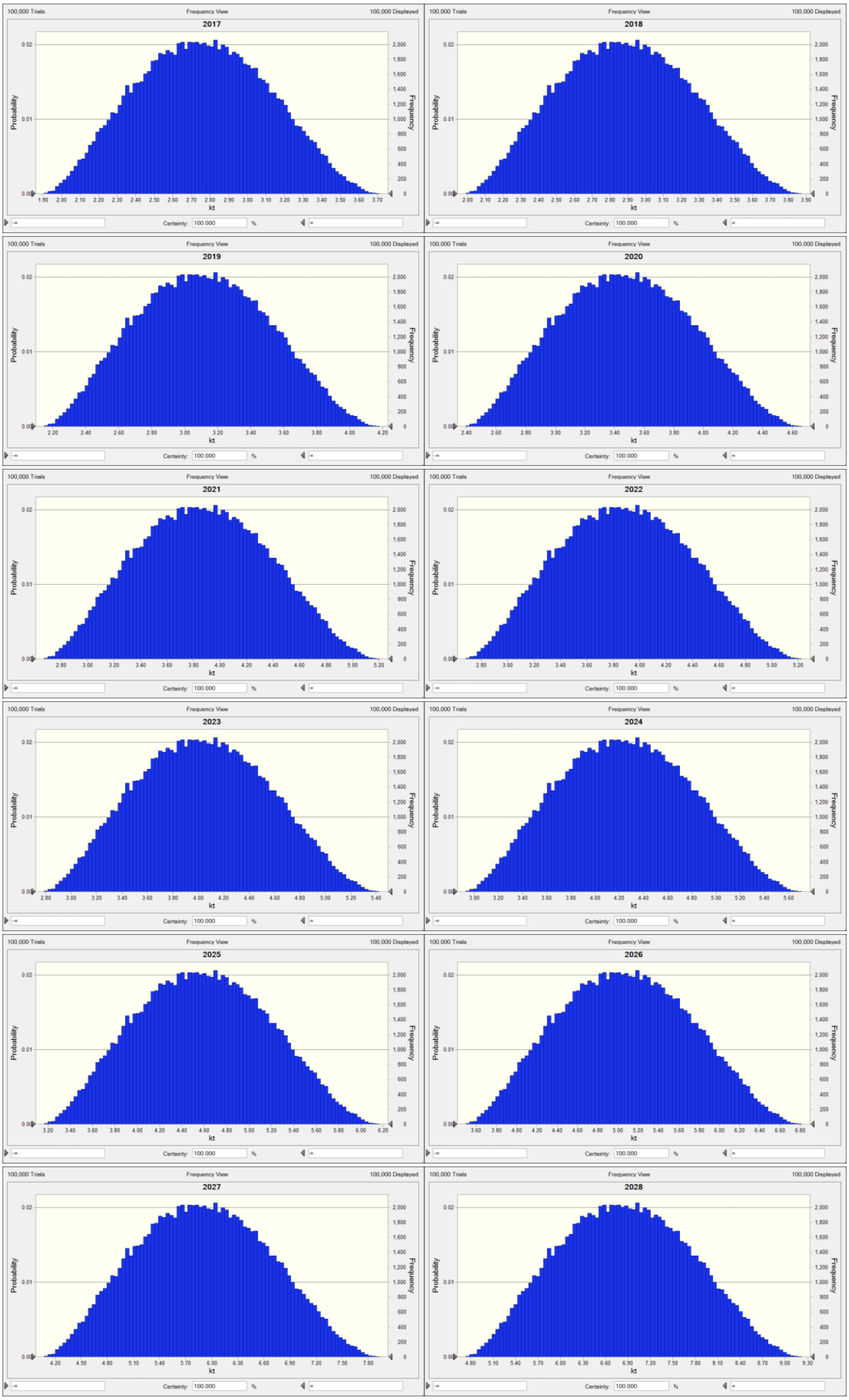
100,000 Trials
Frequency View
100,000 Displayed
2017
2018
2019
2020
2021
2022
2023
2024
2025
2026
2027
2028
Probability
Frequency
kt
Certainty: 100.000 %

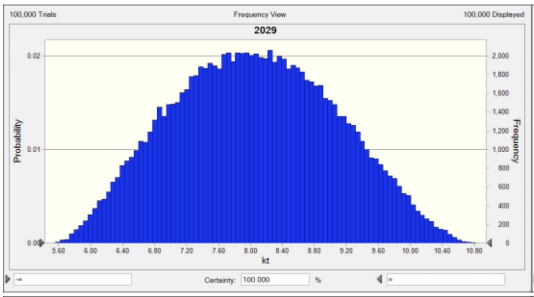
100,000 Trials
Frequency View
100,000 Displayed
2029
Probability
Frequency
kt
Certainty: 100.000 %

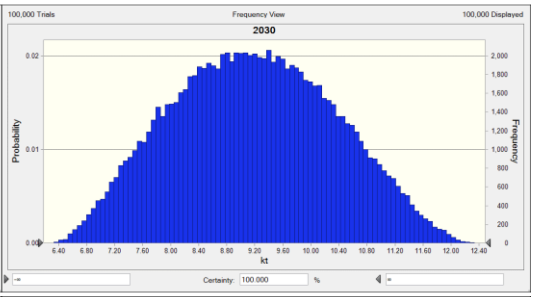
100,000 Trials
Frequency View
100,000 Displayed
2030
Probability
Frequency
kt
Certainty: 100.000 %

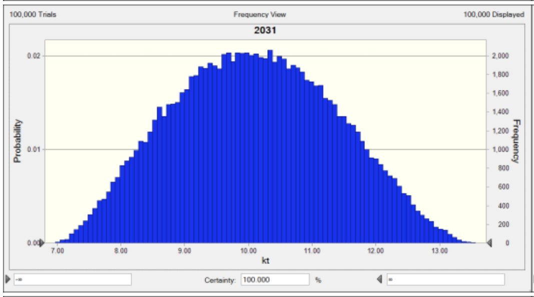
100,000 Trials
Frequency View
100,000 Displayed
2031
Probability
Frequency
kt
Certainty: 100.000 %

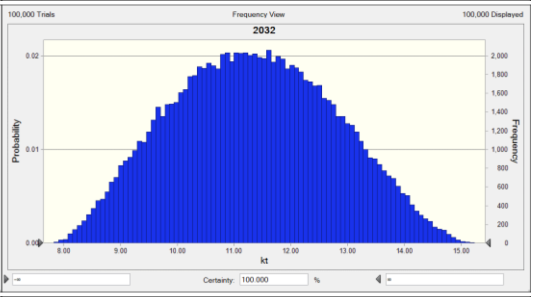
100,000 Trials
Frequency View
100,000 Displayed
2032
Probability
Frequency
kt
Certainty: 100.000 %

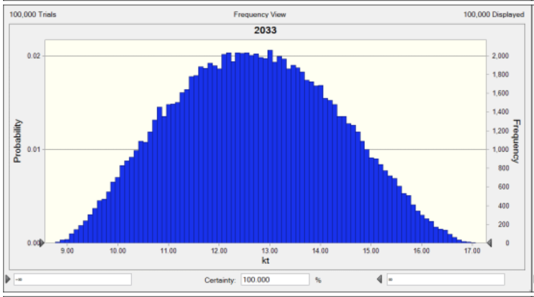
100,000 Trials
Frequency View
100,000 Displayed
2033
Probability
Frequency
kt
Certainty: 100.000 %

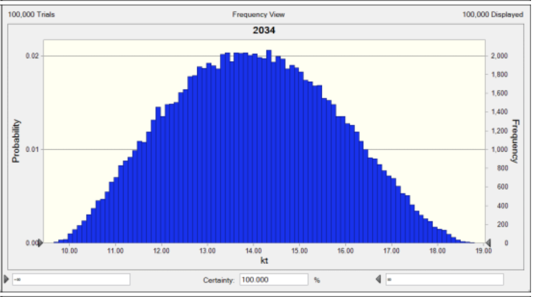
100,000 Trials
Frequency View
100,000 Displayed
2034
Probability
Frequency
kt
Certainty: 100.000 %

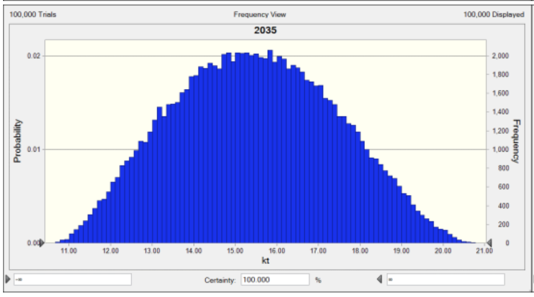
100,000 Trials
Frequency View
100,000 Displayed
2035
Probability
Frequency
kt
Certainty: 100.000 %

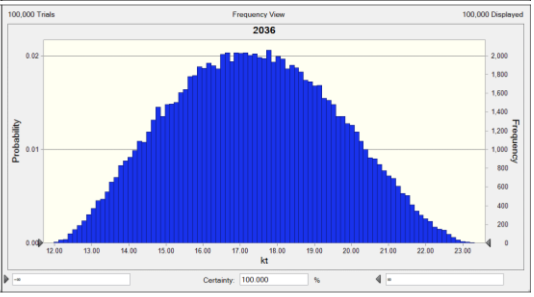
100,000 Trials
Frequency View
100,000 Displayed
2036
Probability
Frequency
kt
Certainty: 100.000 %

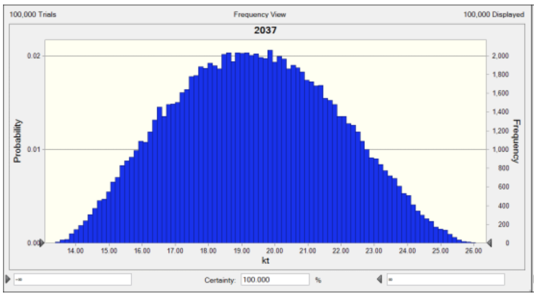
100,000 Trials
Frequency View
100,000 Displayed
2037
Probability
Frequency
kt
Certainty: 100.000 %

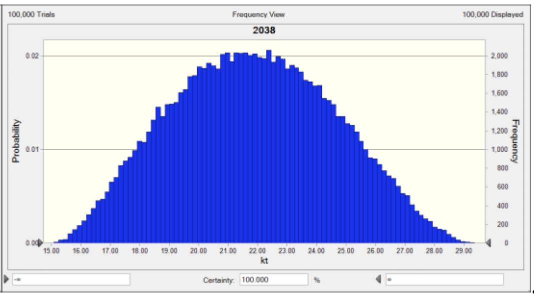
100,000 Trials
Frequency View
100,000 Displayed
2038
Probability
Frequency
kt
Certainty: 100.000 %

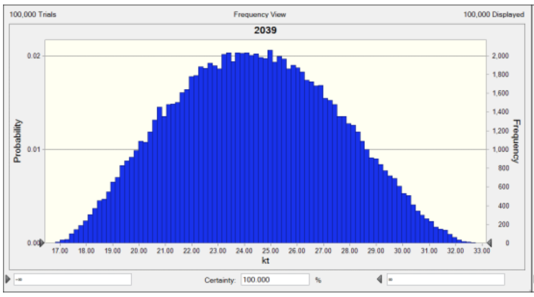
100,000 Trials
Frequency View
100,000 Displayed
2039
Probability
Frequency
kt
Certainty: 100.000 %

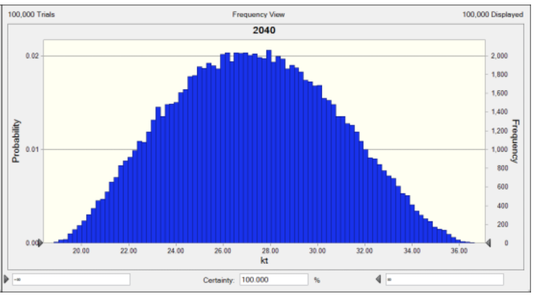
100,000 Trials
Frequency View
100,000 Displayed
2040
Probability
Frequency
kt
Certainty: 100.000 %

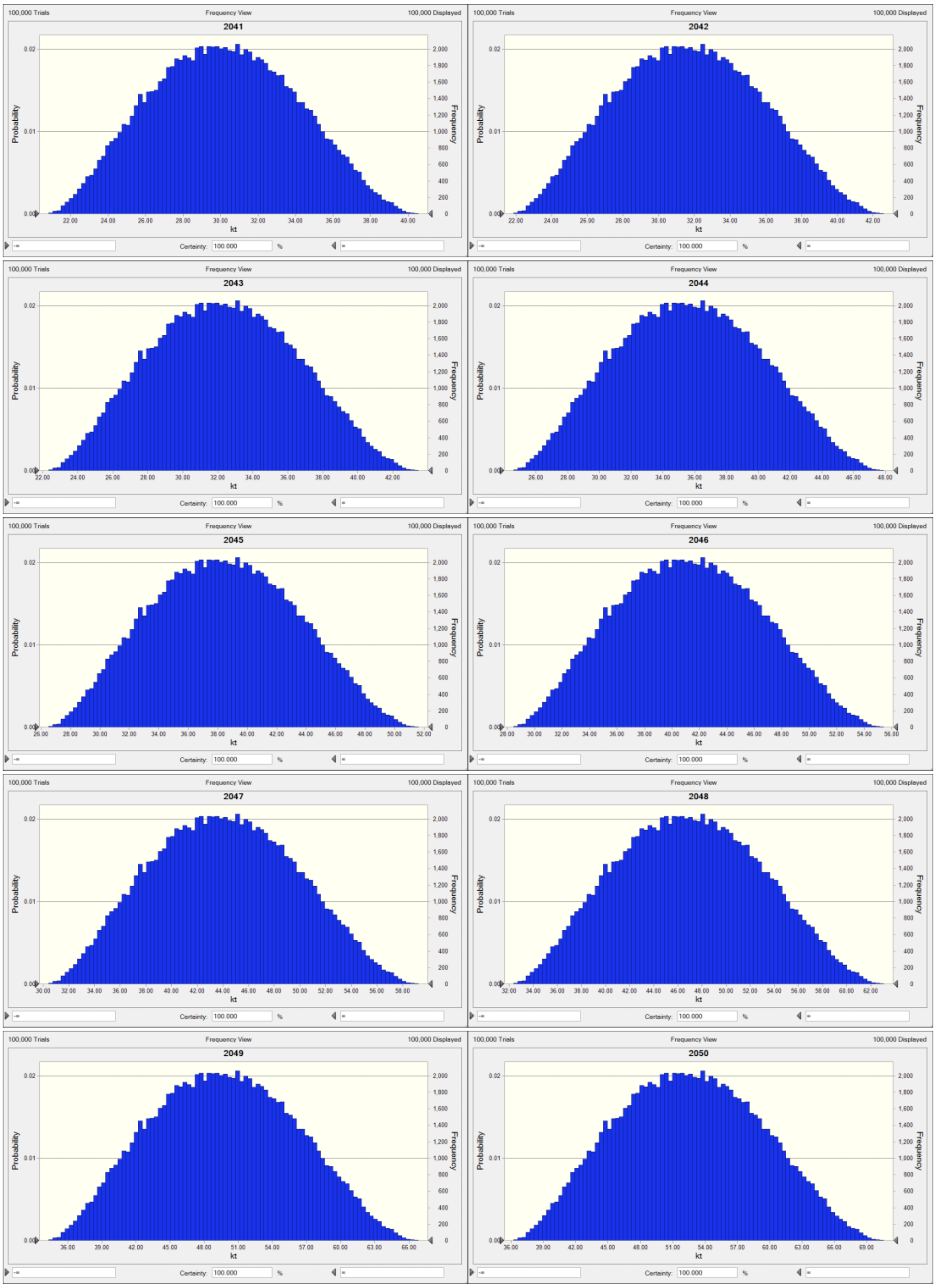

2041
2042
2043
2044
2045
2046
2047
2048
2049
2050
100,000 Trials
Frequency View
100,000 Displayed
Probability
Frequency
kt
Certainty: 100.000 %

f GAA

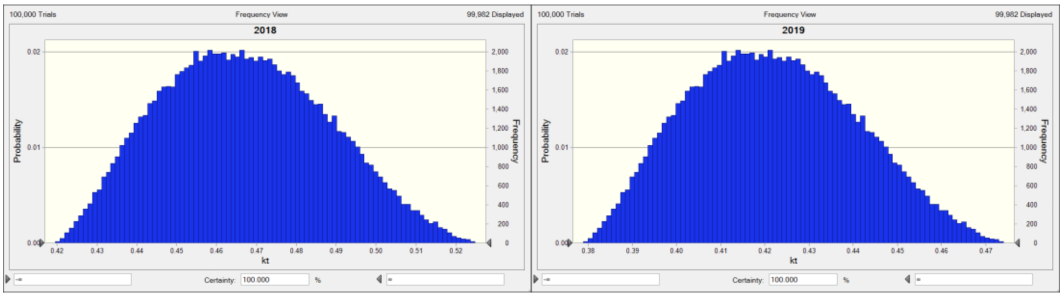

2018
2019
100,000 Trials
Frequency View
99,982 Displayed
Probability
Frequency
kt
Certainty: 100.000 %

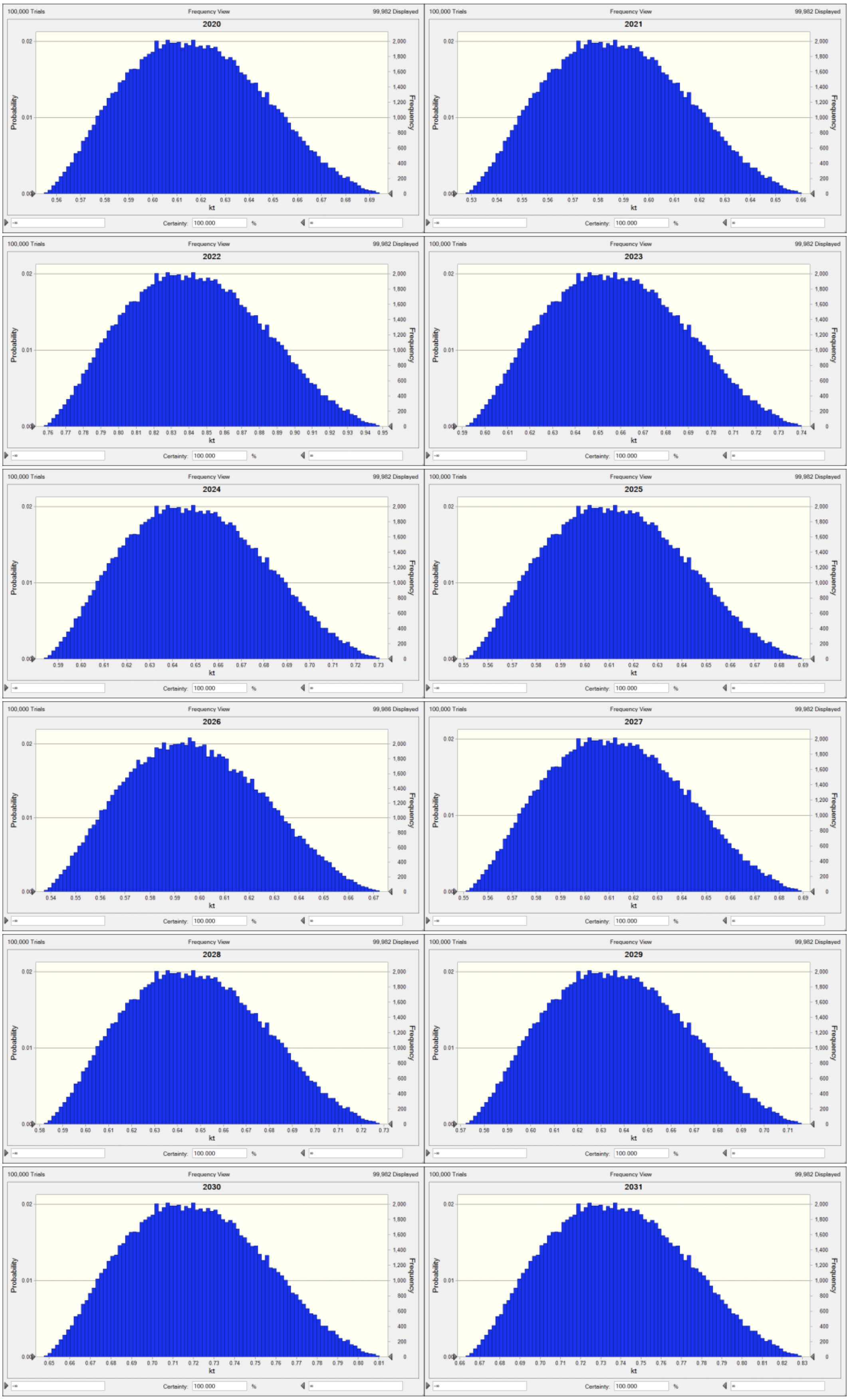
100,000 Trials
Frequency View
99,982 Displayed
2020
2021
2022
2023
2024
2025
99,986 Displayed
2026
2027
2028
2029
2030
2031
Probability
Frequency
kt
Certainty: 100.000 %

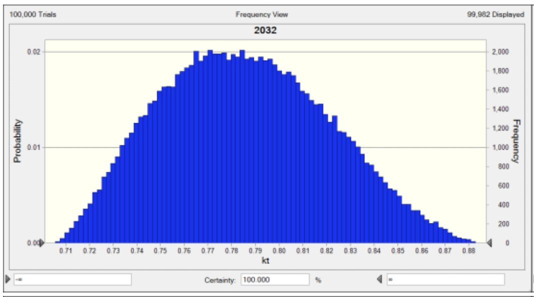
100,000 Trials
Frequency View
99,982 Displayed
2032
Probability
Frequency
kt
Certainty: 100.000 %

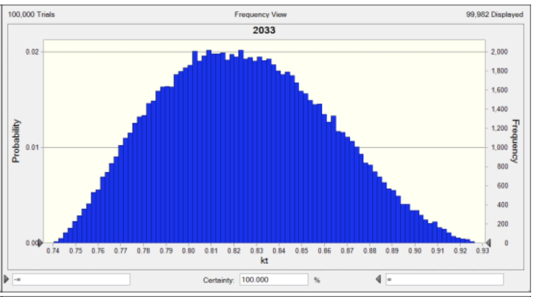
100,000 Trials
Frequency View
99,982 Displayed
2033
Probability
Frequency
kt
Certainty: 100.000 %

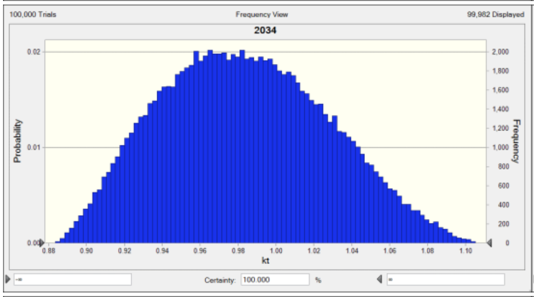
100,000 Trials
Frequency View
99,982 Displayed
2034
Probability
Frequency
kt
Certainty: 100.000 %

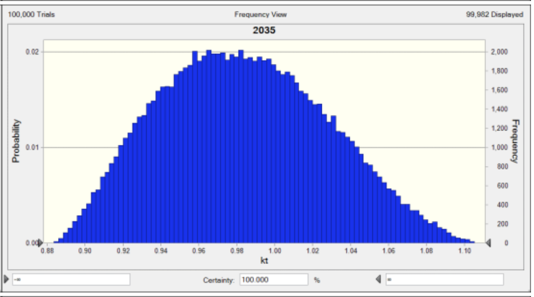
100,000 Trials
Frequency View
99,982 Displayed
2035
Probability
Frequency
kt
Certainty: 100.000 %

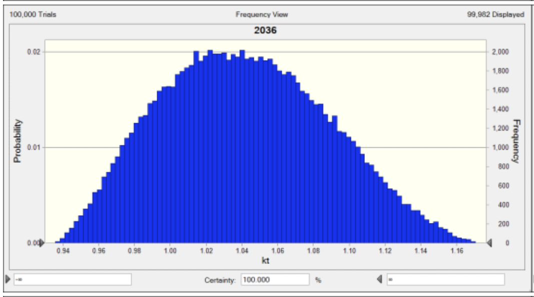
100,000 Trials
Frequency View
99,982 Displayed
2036
Probability
Frequency
kt
Certainty: 100.000 %

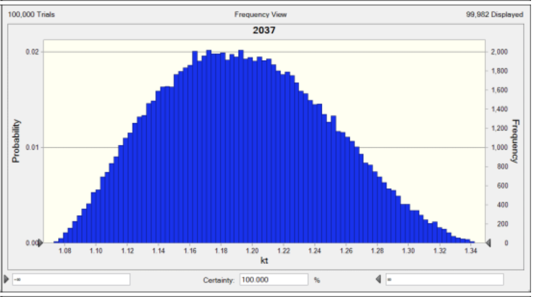
100,000 Trials
Frequency View
99,982 Displayed
2037
Probability
Frequency
kt
Certainty: 100.000 %

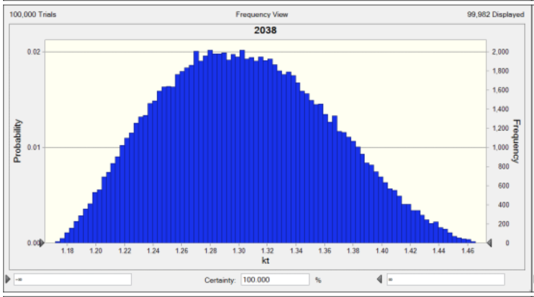
100,000 Trials
Frequency View
99,982 Displayed
2038
Probability
Frequency
kt
Certainty: 100.000 %

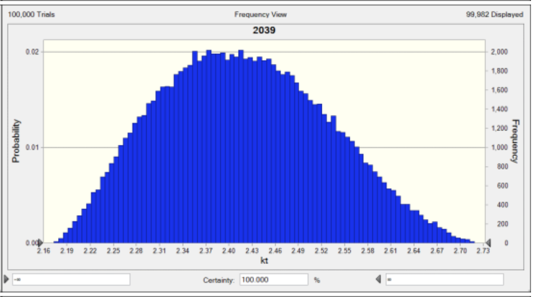
100,000 Trials
Frequency View
99,982 Displayed
2039
Probability
Frequency
kt
Certainty: 100.000 %

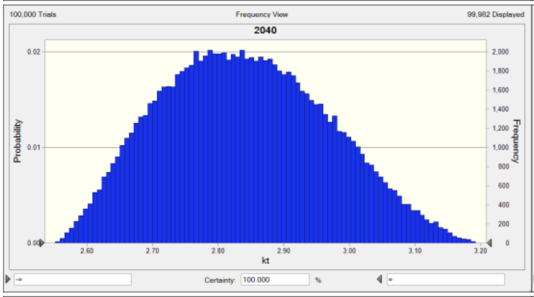
100,000 Trials
Frequency View
99,982 Displayed
2040
Probability
Frequency
kt
Certainty: 100.000 %

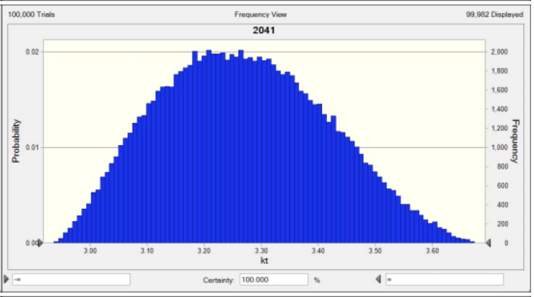
100,000 Trials
Frequency View
99,982 Displayed
2041
Probability
Frequency
kt
Certainty: 100.000 %

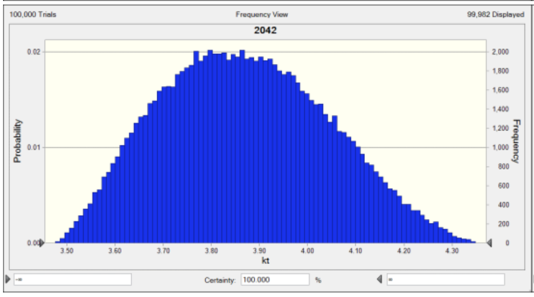
100,000 Trials
Frequency View
99,982 Displayed
2042
Probability
Frequency
kt
Certainty: 100.000 %

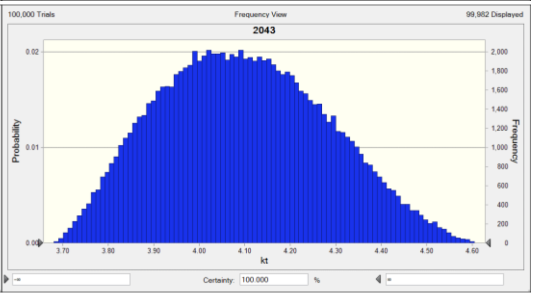
100,000 Trials
Frequency View
99,982 Displayed
2043
Probability
Frequency
kt
Certainty: 100.000 %

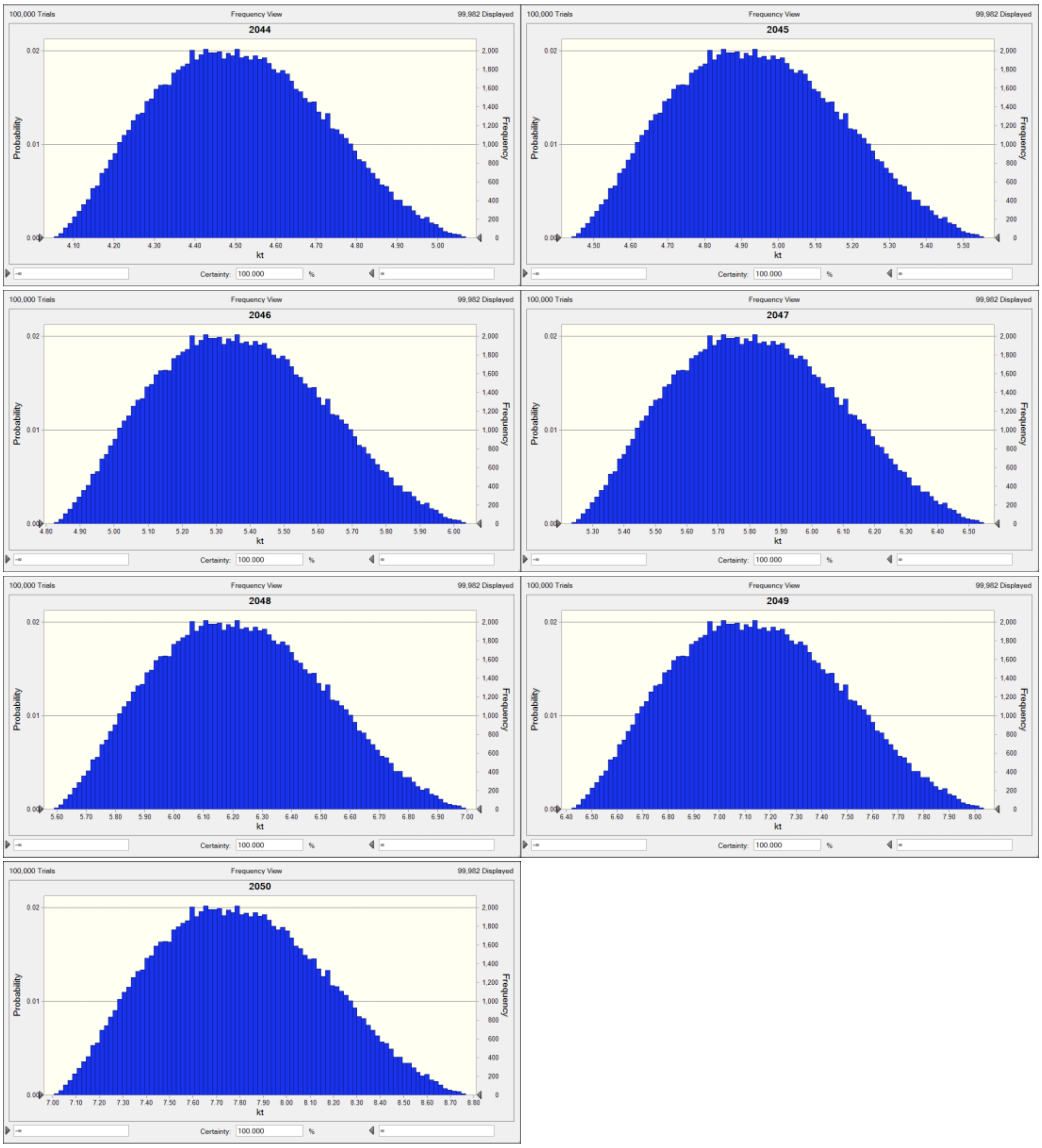

Supplementary Fig. 6 Monte Carlo Simulation for RPC, RL, RW, HST, LMA and GAA

Supplementary Note 1 The abbreviations and acronyms of main vocabulary

ELPV end-of-life private vehicle

EoL end-of-life

GAA general aviation aircraft

HST high speed train

LMA large and medium aircraft

LPTV large-scale public transport vehicle

MFA material flow analysis

RL railway locomotive

RPC railway passenger car

RW railway wagon

WEEE waste electrical and electronic equipment

WLPTV waste large-scale public transport vehicle

WWC waste wiring & cable

Supplementary references

1. Wang C. Macro prospect analysis of high-end equipment industry. *Economic Research Guide* **30**, 26-27 (2017) (in Chinese).

2. Tan Q, Li J. A study of waste fluorescent lamp generation in mainland China. *J. Cleaner Prod.* **81**, 227-233 (2014)

3. Li B, Yang J, Lu B, Song X. Estimation of retired mobile phones generation in China: A comparative study on methodology. *Waste Manage.* **35**, 247-254 (2015)

4. Liu Z, Xu Z, Huang H, Li B. A study of waste liquid crystal display generation in mainland China. *Waste Manag Res* **34**, 58-66 (2016)

5. Song Q, *et al.* Measuring the generation and management status of waste office equipment in China: a case study of waste printers. *J. Cleaner Prod.* **112**, 4461-4468 (2016)

6. Matthews HS, Mcmichael FC, Hendrickson CT, Hart DJ. Disposition and End-of-Life Options for Personal Computers. *US*, (2014)

7. Araujo MG, Magrini A, Mahler CF, Bilitewski B. A model for estimation of potential generation of waste electrical and electronic equipment in Brazil. *Waste Manage.* **32**, 335-342 (2012)

8. Zeng X, Li J, Ren Y. Prediction of various discarded lithium batteries in China. *IEEE*, (2012)

9. Yedla S. Development of a methodology for electronic waste estimation: A material flow analysis-based SYE-Waste Model. *Waste Manage. Res.*, (2015)

10. Walk W. Forecasting quantities of disused household CRT appliances--a regional case study approach and its application to Baden-Württemberg. *Waste Manage.* **29**, 945-951 (2009)

11. Navarro-Esbr J, Diamadopoulos E, Ginestar D. Time series analysis and forecasting techniques for municipal solid waste management. *Resources Conservation & Recycling* **35**, 201-214 (2002)

12. Shan-shan, Chung. Projection of waste quantities: The case of e-waste of the People's Republic of China. *Waste Manage. Res.*, (2011)

13. Huang H, Tong X, Cai Y, Tian H. Re-Examination on Estimation Methods for WEEE Generation in China. *Ecol. Econ.* **35**, 211-216+229 (2019) (in Chinese).

14. He Y, Liao X, Tian H. Research on the Measurement Methods and Result Analysis of Theoretical Scrap Quantity of Domestic Appliance in China. *Appl. Technol.*, 76-77 (2010) (in Chinese).

15. Mass of large-scale public transport vehicle, https://www.docin.com/p-2345769054.html (2020).

16. Woidasky J, Klinke C, Jeanvré S. Materials Stock of the Civilian Aircraft Fleet. *Recycling* **2**, 21-29 (2017)

17. Chen W. Forecasting negative population growth and population ageing in China. *J. Soc. Sci.*, (2022) (in chinese).

18. Ciacci L, Vassura I, Cao Z, Liu G, Passarini F. Recovering the "new twin": Analysis of secondary neodymium sources and recycling potentials in Europe. *Resources Conservation and Recycling* **142**, 143-152 (2019)

19. Asmatulu E, Twomey J, Overcash M. Evaluation of recycling efforts of aircraft companies in Wichita. *Resour. Conserv. Recy.* **80**, 36-45 (2013)

20. Sue G, John, D., Jeannie, G. *Report on the Environmental Benefits of Recycling*. Bureau of International Recycling (2016).

21. Zhang A. Rare earth has broad application space in rail transit. *Rare Earth Inform.*, 34-37 (2019) (in Chinese).

22. Zeng X, Gong R, Chen W, Li J. Uncovering the Recycling Potential of "New" WEEE in China. *Environ. Sci. Technol.* **50**, 1347-1358 (2016)

23. Lifespan database, https://www.nies.go.jp/lifespan/isic_search_e.php (2022).

24. Panchal R, Singh A, Diwan H. Economic potential of recycling e-waste in India and its impact on import of materials. *Resour. Policy* **74**, 102264 (2021)

25. Price of aluminum, https://www.mining.com/markets/commodity/aluminum/ (2021).

26. Price of neodymium, https://www.ac-rei.org.cn/module/d611b22f-9ba5-4e84-915d-2d6763f46e26 (2021).
