# Supplementary material for: China’s recycling potential of large-scale public transport vehicles and its implications
Source: Commun Eng. 2023 Aug 7;2:56. doi: 10.1038/s44172-023-00106-y (PMC10956039; doi:10.1038/s44172-023-00106-y)
Supplement: Supplementary file 3 — Description of Additional Supplementary Files [file 44172_2023_106_MOESM3_ESM.pdf]

Description of Additional Supplementary Files

File name: Supplementary Data 1

Description: The supplementary Data 1 shows the generation and growth rate of waste electrical and electronic equipment (WEEE) in China between 2010 and 2050.

File name: Supplementary Data 2

Description: The supplementary Data 2 shows the generation and growth rate of end-of-life private vehicle (ELPV) in China between 2010 and 2050.

File name: Supplementary Data 3

Description: The supplementary Data 3 shows the generation and growth rate of waste large-scale public transport vehicle (LPTV) in China between 2000 and 2050 .

File name: Supplementary Data 4

Description: The supplementary Data 4 shows the growth rates of WEEE, ELPV, and waste LPTV from 2011 to 2050.
